# Identification of Hanks-Type Kinase PknB-Specific Targets in the *Streptococcus thermophilus* Phosphoproteome

**DOI:** 10.3389/fmicb.2019.01329

**Published:** 2019-06-19

**Authors:** Céline Henry, Lucia Haller, Mélisande Blein-Nicolas, Michel Zivy, Alexis Canette, Morgane Verbrugghe, Christine Mézange, Mylène Boulay, Rozenn Gardan, Samantha Samson, Véronique Martin, Gwenaëlle André-Leroux, Véronique Monnet

**Affiliations:** ^1^Micalis Institute, PAPPSO, INRA, AgroParisTech, Université Paris-Saclay, Jouy-en-Josas, France; ^2^Micalis Institute, ComBac, INRA, AgroParisTech, Université Paris-Saclay, Jouy-en-Josas, France; ^3^PAPPSO, GQE – Le Moulon, INRA, Université Paris-Sud, CNRS, AgroParisTech, Université Paris-Saclay, Gif-sur-Yvette, France; ^4^Micalis Institute, MIMA2, INRA, AgroParisTech, Université Paris-Saclay, Jouy-en-Josas, France; ^5^MaIAGE, INRA, Université Paris-Saclay, Jouy-en-Josas, France

**Keywords:** protein phosphorylation, *Streptococcus thermophilus*, Hanks-type kinase, proteomics, cellular division

## Abstract

Protein phosphorylation especially on serine/threonine/tyrosine residues are frequent in many bacteria. This post-translational modification has been associated with pathogenicity and virulence in various species. However, only few data have been produced so far on generally recognized as safe bacteria used in food fermentations. A family of kinases known as Hanks-type kinases is suspected to be responsible for, at least, a part of these phosphorylations in eukaryotes as in bacteria. The objective of our work was to establish the first phosphoproteome of *Streptococcus thermophilus*, a lactic acid bacterium widely used in dairy fermentations in order to identified the proteins and pathways tagged by Ser/Thr/Tyr phosphorylations. In addition, we have evaluated the role in this process of the only Hanks-type kinase encoded in the *S. thermophilus* genome. We have constructed a mutant defective for the Hanks type kinase in *S. thermophilus* and established the proteomes and phosphoproteomes of the wild type and the mutant strains. To do that, we have enriched our samples in phosphopeptides with titane beads and used dimethyl tags to compare phosphopeptide abundances. Peptides and phosphopeptides were analyzed on a last generation LC-MS/MS system. We have identified and quantified 891 proteins representing half of the theoretical proteome. Among these proteins, 106 contained phosphorylated peptides. Various functional groups of proteins (amino acid, carbon and nucleotide metabolism, translation, cell cycle, stress response, …) were found phosphorylated. The phosphoproteome was only weakly reduced in the Hanks-type kinase mutant indicating that this enzyme is only one of the players in the phosphorylation process. The proteins that are modified by the Hanks-type kinase mainly belong to the divisome.

## Introduction

Protein post-translational modifications are frequent and diverse in bacteria. These modifications, not genetically encoded, affect protein conformation, activity and function. They allow quick sensing and responses to environmental changes and participate in transduction networks. Among described protein modifications, phosphorylation is the most documented. It targets several amino acids, but serine, threonine and tyrosine phosphorylations are the most studied, as they are stable enough to be analyzed by mass spectrometry. Ser/Thr/Tyr phosphorylation has frequently been associated with bacterial pathogenicity in various species. Kinases, belonging to a main structural family, first described by Hanks et al. (1988) and widespread in both eukaryotes and procaryotes, have emerged as potentially responsible for these phosphorylations (Cousin et al., 2013). Stancik et al. (2018) recently proposed to call these kinases ‘Hanks-type kinases.’

We were, in this work, interested in the role of Ser/Thr/Tyr protein phosphorylation in *Streptococcus thermophilus*, that is the only GRAS (Generally Recognized As Safe) species in the Streptococcaceae family, which contains mostly pathogenic and commensal species. *S. thermophilus* is widely and worldwide used as a technological starter bacterium in various milk product fermentations. *S. thermophilus* also belongs to the so-called group of lactic acid bacteria merging diverse bacterial species characterized by their ability to produce lactic acid during fermentation and associated to plant, meat and dairy products. During technological processes, *S. thermophilus* has to adapt to various nutrition and physical-chemical stresses that require rapid adjustments. We already demonstrated that streptococci have developed specific cell-cell communication and regulation systems based on peptide pheromones to control specific functions (Fleuchot et al., 2011). In this study, we investigated another way for *S. thermophilus* to regulate specific pathways namely post-translational protein modifications and more specifically protein serine/threonine/tyrosine phosphorylation. We aimed at identifying the pathways that are modulated by phosphorylation in this bacterium.

Phosphoproteomic studies are very scare in lactic acid bacteria and exclusively concern serine/threonine/tyrosine residues. A pioneer study on the Ser/Thr/Tyr phosphoproteome of *Lactococcus lactis*, another dairy technological starter identified 73 phosphorylation sites and multiple multi-phosphorylated proteins. The phosphorylated proteins are well represented in house-keeping and glycolytic pathways (Soufi et al., 2008). In *Oenococcus oeni*, a bacterium used in grape juice fermentation, 39 phosphorylation sites were identified in 19 proteins (Napoli et al., 2014). Finally, a study on the effect of acid stress on protein phosphorylation in *Lactobacillus rhamnosus* GG identified 15 proteins from central pathways and especially from carbon metabolism and glycolysis that are phosphorylated in response to acid (Koponen et al., 2012).

In streptococci, global Ser/Thr/Tyr phosphoproteomic studies exclusively concern the model pathogenic species, *S. pneumoniae.* Phosphoproteomics of this bacterium, established in exponentially growing state, allowed to identify 84 phosphorylated proteins. The latter are involved in carbon, nitrogen, nucleic acid metabolisms, cell division but also targets proteins of unknown functions (Sun et al., 2010). The kinases involved in Ser/Thr/Tyr phosphorylation in streptococci and lactic acid bacteria are not well identified. *S. pneumoniae, Streptococcus suis* as well as *Streptococcus agalactiae* have one Hanks-type kinase encoding gene in their genomes. The *S. pneumoniae* Hanks-type kinase is localized at the division site and involved in cell division control (Beilharz et al., 2012). It phosphorylates at least 10 proteins, including cell division proteins, proteins of unknown functions and the Hanks-type kinase itself (Silvestroni et al., 2009). The Hanks-type kinase from *S. suis* is involved in the phosphorylation of 12 proteins associated with cell cycle, glycolysis and translation and participates in pathogenicity in mice (Zhang et al., 2017). The one of *S. agalactiae* phosphorylates at least six proteins, including a pyrophosphatase. It also modulates purine biosynthesis and growth (Rajagopal et al., 2003, 2005). From these studies, realized in different conditions and with different experimental protocols, it remains difficult to predict the set of proteins that are targets of Hanks-type kinases. In addition, the relative role of the Hanks-type kinase in the whole Ser/Thr/Tyr process is not well established and the other kinases acting in the process are not identified. Production of new bacterial phosphoproteomics studies will help to identify the actors of phosphorylation and the basis of their specificities. The objectives of the present work were to investigate the Ser/Thr/Tyr phosphorylation process and its role in *S. thermophilus*, the only streptococcal species used in food fermentation, and to assess the specific role of the only predicted Hanks-type kinase, named PknB, encoded in its genome.

We showed that the *S. thermophilus* Ser/Thr/Tyr phosphoproteome is of the same order of magnitude than the other streptococci ones as peptides belonging to 106 proteins from various metabolic pathways were found phosphorylated in one bacterial growth condition. We demonstrated that the Hanks-type kinase, named PknB in *S. thermophilus*, targets the divisome and is only one of the players in the Ser/Thr/Tyr phosphorylation process. Consistent with these results, the *pknB* deletion mutant exhibits a clear phenotype with longer whimsical chains and affected division process. Finally, we identified two putative kinases that could, with PknB, phosphorylate proteins in *S. thermophilus*.

## Materials and Methods

### *In silico* Screening of the Whole Genome of *Streptococcus thermophilus* LMD9 and Identification of Structural Homologs to PknB

The whole genome was downloaded from the NCBI^1^ and each of the 1681 coding genes was split into a single fasta sequence. Then the HHsuite (Söding et al., 2005), dedicated to homology detection and structure prediction, was used to predict sequences that could share the 3D fold of the kinase domain of PknB from *Mycobacterium tuberculosis* that serves as template. For each fasta sequence of the genome, a multiple sequence analysis (MSA) was performed using HHblits, and a matrix of similarity score using Hidden Markov Model was calculated. Each profile was then compared to the HMM profile of the PknB_Mtb_ kinase template. Only were kept for sequence/structure/function analysis the sequences that associated a probability above 95% to share the PknB_Mtb_ kinase fold, with a size above 100 amino-acid residues and an e-value below 1.e^−15^.

### Homology Modeling of PknB Catalytic Domain and PASTA Domains

The catalytic domain of PknB_St_ (residues 3–274) was homology-modeled using the model-building software Modeller (version 9.18) (Sali and Blundell, 1993). The crystal structure of the catalytic domain of PknB from *M. tuberculosis* (PknB*_Mtb_* 1O6Y pdb id) in complex with AMP-PCP served as 3D template (Ortiz-Lombardia et al., 2003). Hundred models were generated. A final model of PknB_St_ was selected upon the lowest score function values (DOPE score) and best stereochemistry, checked by Molprobity (Chen et al., 2010). Subsequently, a molecule of ATP was docked in the binding pocket of this model of PknB_St_, with Autodock tools, using a grid box, centered on the ATP-binding pocket, and the genetic algorithm of Lamarck (Morris et al., 2009). To validate this procedure, a crystal molecule of AMP-PCP was previously redocked in the ATP-binding pocket of PknB_Mtb_ using similar gridbox and algorithm. Similarly, the three PASTA domains (residues 363–579) of the extracellular domain (ECD) of PknB_St_ were homology modeled using Modeller, from the PASTA-domain crystal templates of PknB_Mtb_ (pdb id 5E12, Prigozhin et al., 2016) and PASTA from Ser/Thr protein kinase (STK1) of *Staphylococcus aureus* (pdb id 3M9G, Paracuellos et al., 2010) as 3D templates. A final model of PASTA-ECD_St_ was chosen upon lowest score functions and subsequently checked by Molprobity. Using HHpred^2^ (Zimmermann et al., 2018) the residues 275–362 that correspond to the juxtamembrane and the transmembrane segments, showed no structural homolog so that this portion could not be modeled. For the same reason, the C-terminal stretch of 44 residues could not be modeled. All the selected models were visually inspected and figures generated are rendered with Pymol 2.0.7. (Schrödinger, LLC).

### Construction of a Δ*pknB* Mutant Strain

We used the overlapping PCR method to delete a part of the PknB encoding gene (STER_1392; STER_RS06845) in the LMD-9 strain (Makarova et al., 2006). Briefly, we generated 3 fragments by PCR using the oligonucleotides described in Table 1. The first fragment (1350 bp) was the kanamycin (aphaA3) cassette, which was amplified with oligonucleotides AphaA3-F and AphaA3-R using the plasmid pKa as template (Débarbouillé et al., 1990). The two other fragments (450 bp each), called F1 and F2, were located upstream and downstream the *pknB* gene and were amplified with oligonucleotides 1–2 and 3–4, respectively, using chromosome DNA from *S. thermophilus* LMD-9 as template (Table 1). Thanks to the presence of extensions compatible with the apha3 cassette in oligonucleotides 2 and 3, the three fragments aphaA3, F1 and F2 were fused together in one PCR. The resulting PCR fragment was used to transform *S. thermophilus* taking advantage of its natural competence in chemically defined medium (Letort and Juillard, 2001). The clones in which a double recombination occurred and which were kanamycin resistant were selected and checked for integration of the aphaA3 cassette at the *pknB* locus with oligonucleotides 5 and 6 (2431 bp in the mutant) (Table 1). One clone was kept, verified by sequencing and stored under the number TIL1509.

**Table 1:** Strains and oligonucleotides.

**Oligonucleotides used to construct the *pknB* mutant**
AphaA3-F: CCAGCGAACCATTTGAG
AphaA3-R: GTTGCGGATGTACTTCAG
1: ATGCTGTGATTTTCGCTC
2: GACATCTAATCTTTTCTGAAGTACATCCGCAACGGATTCGATAACGTCCAGCA
3: ATAATCTTACCTATCACCTCAAATGGTTCGCTGGAGTACGACACAATCGTCGTC
4: CAAAAATCCGCTCCCGAA
5: TAAAGGAATGGGAACGAC
6: GACCAATTCAACGTGAGA
**Positions of the oligonucleotides and of the PCR fragments**
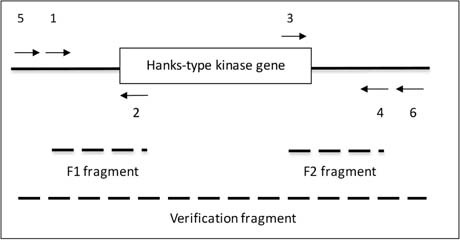

### Culture Conditions

*S. thermophilus* strain LMD-9 (WT) and its Δ*pknB* mutant (Mut) were grown in M17 medium (Difco) supplemented with 10 g.L^−1^ lactose (M17Lac) at 42°C and stopped in the exponential growth phase when pH reached 6.20 (after 2,5 h for the wild type and 3 h for the mutant). Agar (1.5%) was added to the medium as appropriate. When required, kanamycin (1 mg.ml^−1^) was added to the medium. The optical density at 600 nm of cultures was measured with an Uvikon 931 spectrophotometer (Kontron). pH was also followed using a pH-meter. For proteomics experiments, four independent replicates of the culture of each strain (WT 1–4, Mut 1–4) were performed, so that the total number of samples was eight.

A stress test was realized as follows: cultures were grown in triplicates up to stationary phase. Numerations were done at this time, and again after one night freezing at −20°C and defreezing at 25°C. Bacterial counts were compared for each strain, before and after freezing.

### Scanning Electron Microscopy Analysis

For two replicates of WT and Δ*pknB* mutant, one 40 μL drop of fresh M17 liquid culture (collected at exponential phase; OD_600nm_ = 0.7) was deposited onto a sterile aluminum coupon (10 mm diameter, sterilized just before use by sonication in ethanol and dried during UV exposure) placed into one well of a 24-wells polystyrene plate. Sedimentation of bacteria lasted 1.5 h. Samples were fixed via careful immersion in a solution of 2.5% (v/v) glutaraldehyde in 0.1 M sodium cacodylate buffer at pH 7.4 and room temperature (RT) followed by overnight waiting time at 4°C. Samples were then washed three times for 5 min with 0.1 M sodium cacodylate buffer. Samples were dehydrated with increasing concentrations of ethanol at room temperature (50–70–90%-3 × 100%, v/v with ultra-pure water) for 10 min at each step. Samples were critical-point dried (Quorum Technologies K850, Elexience, France) at 70 bar and 37°C with liquid CO_2_ as the transition fluid and then depressurized slowly (400 cm^3^ min^−1^). Each aluminum support carrying the sample was then mounted on an aluminum stub with double-sided carbon tape. Samples were sputter-coated (Polaron SC7640, Elexience, France) in Ar plasma with Pt (approximately 30 nm thick) at 10 mA and 0.8 kV over a duration of 200 s. Observations were performed in a field-emission SEM (Hitachi S4500, Elexience, France) in high vacuum, with a secondary electron low detector, at 2 kV and 16 mm working distance, on the MIMA2 imaging platform (INRA^3^).

### Protein Extraction

At an OD_600_ of 0.7, bacteria were harvested by centrifugation (10 min at 6,000 *g*, 4°C). The cell pellet was washed twice with phosphate buffered saline (200 mM, pH 6.4) and resuspended in lysis buffer (Tris-HCl 50 mM pH 8.0; 50 μl/ml Protease Inhibitor Cocktail P8465, Sigma; 10 μl/ml Halt Phosphatase Inhibitor Cocktail 78420, Thermo Scientific). Cells were lysed using a Cell Disruptor (One Shot 0.75 kW, Constant Systems Ltd.) and non-lysed cells as well as cell debris were separated from the protein-containing supernatant by centrifugation (15 min at 20,000 *g*, 4°C). The protein content was measured using the Bradford assay (Bradford, 1976) and the protein extract was used for the proteome as well as for the phosphoproteome analyses.

### Sample Preparation for Proteome Analysis

**FIGURE 1:**
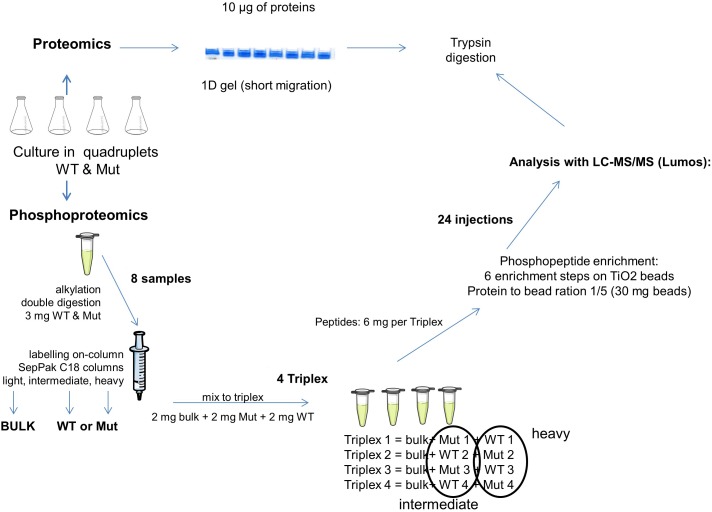
Schematic representation of the proteomics and phosphoproteomics pipeline used in this study. Bulk, mixture of all samples; WT, samples from the wild type strain; Mut, samples from the mutant strain.

For the proteome analysis (Figure 1), 10 μg of protein extract were used for a short migration 1D gel electrophoresis (NuPAGE^®^ 4-12% Bis-Tris Gel, novex). Proteins were visualized using Coomassie G-250 (SimplyBlue^TM^ SafeStain, Invitrogen) and the whole colored part of each lane was cut into small pieces. The gel pieces were destained using Solvent A (10% v/v acetic acid, 40% v/v ethanol) and Solvent B (50% v/v 50 mM ammonium bicarbonate, 50% v/v acetonitrile). The proteins contained in the gel were reduced by 10 mM dithiothreitol (Sigma) and alkylated by 55 mM iodoacetamide (Sigma). The proteins were digested with 200 ng trypsin (Promega) and afterwards extracted using a solution of 0.5% v/v trifluoroacetic acid and 50% v/v acetonitrile. The peptides were dried completely using a concentrator (Savant^TM^ SPD121D, Thermo Fisher Scientific) and taken up in 50 μl loading buffer (0.08% v/v trifluoroacetic acid, 2% v/v acetonitrile) for LC-MS/MS proteome analysis (4 μl = 800 ng peptide per injection).

#### Peptide Labeling for Phosphoproteome Analysis

For the phosphoproteome analysis (Figure 1), 3 mg of protein extract were denatured using 6 M urea and 2 M thiourea as well as 0.1% w/v of RapiGest (RapiGest SF Surfactant, Waters). Reduction and alkylation were achieved by 5 mM dithiothreitol and 15 mM iodoacetamide. Proteins were digested by Lys-C (Lysyl Endopeptidase^®^, Mass Spectrometry Grade, Wako; enzyme/substrate ratio of 1:50 w/w) and, after a dilution to 1 M urea, by trypsin (Sequencing Grade Modified Trypsin, Promega; 1:50 w/w). The reaction was terminated with 0.1% v/v trifluoroacetic acid.

The samples were labeled on SepPak C18 cartridge columns 3 cc (Waters) with 7 times 1 ml of their respective labeling solutions during 10 min (Boersema et al., 2009). The solution for light labeling was composed of 90% v/v 50 mM sodium phosphate buffer pH 7.5, 5% v/v formaldehyde 4%, 5% v/v 0.6 M cyanoborohydride. For the intermediate labeling, formaldehyde was replaced with deuterated formaldehyde and for heavy labeling, formaldehyde was replaced with ^13^C and deuterium labeled formaldehyde and cyanoborohydride was replaced with cyanoborodeuteride. The labeled peptides were eluted twice with 500 μl elution buffer (0.6% v/v acetic acid, 80% v/v acetonitrile).

Four triplex containing light-, intermediate-, and heavy-labeled samples in equal proportions (2 mg of peptide per labeling) were assembled. The light sample was the technical control. It was prepared by mixing equal quantities of all samples of the experiment. To eliminate any possible bias due to different labeling efficiencies or binding affinities, the labeling of the different replicates was switched, so that *in fine* two replicates of each strain were labeled “heavy” and the two others were labeled “intermediate” (Figure 1).

#### Phosphopeptide Enrichment

The experimental protocol for phosphoproteomics used in this study involved a titane beads enrichment step. It was lighter and more rapid than a protocol based on SCX chromatography while preserving the same effectiveness (Misra et al., 2014).

The triplexes were concentrated to a volume of 200 μl before being mixed with 555 μl lactic acid, 1,200 μl acetonitrile, 2 μl trifluoroacetic acid and filled up to 2 ml with purified H_2_O (Milli-Q^®^, Merck Millipore). The enrichment was performed in six consecutive steps with 30 mg TiO_2_ beads (Titansphere TiO, 10 μm, GL Sciences) per step. The TiO_2_ beads were first washed with loading solution (80% v/v acetonitrile, 6% v/v trifluoroacetic acid) and then transferred to the sample. The sample-bead mixture was incubated at room temperature for 20 min at 7 rpm. After spinning down (3 min at 4,000 g) the beads, the supernatant was transferred to the beads of the next enrichment step and incubation and spinning down were repeated as above. The beads with the bound phosphopeptides were washed once with Wash solution I (30% v/v acetonitrile, 1% v/v trifluoroacetic acid) and once more with Wash solution II (80% v/v acetonitrile, 1% v/v trifluoroacetic acid) before being transferred to a tip containing a 3M Empore^TM^ phase (Sigma-Aldrich). Elution of the phosphopeptides into a tube containing 20 μl 20% v/v trifluoroacetic acid was completed in 2 steps, first with 2 times 100 μl of the basic Elution solution I (5% v/v ammonium hydroxide, 60% v/v acetonitrile) and then with 50 μl of the acidic Elution solution II (80% v/v acetonitrile, 1% v/v formic acid) under rotation (3 min at 4,000 g). Samples were reduced in a concentrator to a volume of 1 μl and taken up in 30 μl loading buffer (0.08% v/v trifluoroacetic acid, 2% v/v acetonitrile) for LC-MS/MS phosphoproteome analysis. The mass shift caused by light, intermediate and heavy dimethylation was +28, +32, and +36 Da, respectively.

#### Liquid Chromatography – Mass Spectrometry

Mass spectrometry was performed on the PAPPSO platform (MICALIS, INRA, Jouy-en-Josas, France^4^). An Orbitrap Fusion^TM^ Lumos^TM^ Tribrid^TM^ (Thermo Fisher Scientific) coupled to an UltiMate^TM^ 3000 RSLCnano System (Thermo Fisher Scientific) was used.

A 4 μl sample was loaded at 20 μl/min on a precolumn (μ-Precolumn, 300 μm i.d × 5 mm, C18 PepMap100, 5 μm, 100 Å, Thermo Fisher) and washed with loading buffer. After 3 min, the precolumn cartridge was connected to the separating column (Acclaim PepMap^®^, 75 μm × 500 mm, C18, 3 μm, 100 Å, Thermo Fisher). Buffer A consisted of 0.1% formic acid in 2% acetonitrile and buffer B of 0.1% formic acid in 80% acetonitrile.

The proteome runs were executed at 300 nl/min with a linear gradient from 0 to 45% buffer B for 118 min. Including regeneration (98% buffer B), one run took 137 min. Ionization (1.6 kV ionization potential) and capillary transfer (275°C) were performed with a liquid junction and a capillary probe. Peptide ions were analyzed using Xcalibur 3.1.66.10 with the following machine setup in CID (Collision Induced Dissociation) mode: (1) full MS scan in Orbitrap (scan range [m/z] = 400–1500) and (2) MS/MS using CID (35% collision energy) in Ion Trap (AGC target = 4.0 × 10^3^, max. injection time = 300 ms, data type = centroid). Analyzed charge states were set to 2–7, the dynamic exclusion to 60 s and the intensity threshold was fixed at 5.0 × 10^3^.

The peptide separation for the phosphoproteome analysis was achieved at 300 nl/min with a linear gradient from 0 to 35% buffer B for 48 min and 35–45% for 5 min. One run took 65 min, including the regeneration step at 98% buffer B. Ionization (1.6 kV ionization potential) and capillary transfer (275°C) were performed with a liquid junction and a capillary probe (SilicaTip^TM^ Emitter, 10 μm, New Objective). Peptide ions were analyzed using Xcalibur 3.1.66.10. In HCD (Higher-energy Collisional Dissociation) mode the machine settings were as follows: (1) full MS scan in Orbitrap (scan range [m/z] = 400–1600) and (2) MS/MS using HCD (30% collision energy) in Orbitrap (AGC target = 5.0 × 10^4^, max. injection time = 55 ms, data type = centroid). Analyzed charge states were set to 2–5, the dynamic exclusion to 40 s and the intensity threshold was fixed at 10^4^.

The MS proteomics data have been deposited to the ProteomeXchange Consortium via the PRIDE (Vizcaino et al., 2016) partner repository with the dataset identifier PXD011391.

#### Data Analyses

The *S. thermophilus* LMD-9 database (NCBI, version 2010, 1710 entries) was searched by using X!TandemPipeline version 3.4.2 (Langella et al., 2017). In the database used, the proteins where initially annotated using a Blast against the genomes of the strains CNRZ1066 and LMG18311 (which were the two first strains sequenced) with variable e-values which are not always very high and which consequently must be considered carefully.

The proteome identification was run with a precursor mass tolerance of 10 ppm and a fragment mass tolerance of 0.5 Da. Enzymatic cleavage rules were set to trypsin digestion (“after Arg and Lys, unless Pro follows directly after”) and no semi-enzymatic cleavage rules were allowed. The fix modification was set to cysteine carbamidomethylation and methionine oxidation was considered as a potential modification. In a second pass, N-terminal acetylation was added as another potential modification, whereas all other previous settings were retained. The identified proteins were filtered as follows: (1) peptide *E*-value < 0.05 with a minimum of 2 peptides per protein and (2) a protein *E*-value of <10^−4^. In routine analyses, protein identification was associated to a function prediction obtained via an elementary Blast toward two *S. thermophilus* genome sequences (LMG13811 and CNRZ1066). This function prediction need to be carefully considered. A more in-depth Blast was realized against the whole NCBI database for the proteins which were identified as phosphorylated.

For the phosphoproteome identification, the precursor mass tolerance was set at 10 ppm, while the fragment mass tolerance was set at 0.02 Da. The enzymatic cleavage was set to tryptic rules with no semi-enzymatic cleavages being considered. Cysteine carbamidomethylation as well as the mass-increments due to labeling (+28.0313 Da, +32.0564 Da, +36.0756 Da) at the C-terminus and lysine residues were set to static modifications. Methionine oxidation and S/T/Y phosphorylation were considered as potential modifications. A refinement search was performed with the above mentioned parameters and the additional potential modification of N-terminal acetylation. The identified phosphoproteins were filtered as follows: (1) stringent peptide *E*-value < 0.001 with a minimum of 1 peptide per protein and (2) a protein *E*-value of <10^−2^. The labeling efficiency was at 99.7%.

The PAPPSO tool X!TandemPipeline (Langella et al., 2017) works with phosphoislands and identified the best phosphorylation position as well as all possible positions when several phosphorylations are possible within the same peptide.

Proteins and phosphoproteins were quantified by the spectral counting (SC) method. MassChroQ “Black Caiman” version 2.2.1 (Valot et al., 2011) was used for the alignment of retention time. The range for peak detection was set to 10 ppm with a detection threshold ranging from 30,000 to 50,000.

MassChroqR (version 0.3.7), an R package developed by PAPPSO platform^5^ was used to check the quality of data and practice statistical analysis in proteomic. The abundance in number of spectra was modeled using the following generalized mixed model (GLM) with a Poisson distribution as already described (Millan-Oropeza et al., 2017). Protein abundance change were detected by analysis of variance (ANOVA) using a Chi-square test. The obtained *p*-values were adjusted for multiple testing by the Benjamini-Hochberg approach (Benjamini and Hochberg, 1995). Adjusted *P*-values obtained from ANOVA for the proteome was considered significant below a value of 0.05.

For phosphoproteomics, quantification was performed at the peptide level and not at the protein level. In this case, the number of spectra associated to each peptide is generally low (i.e., comprised between 0 and 2), which prevent proper statistical analysis of spectral counting data. We thus focused on the phosphopeptides showing presence/absence variations between the WT and the Mut strains. To ensure robustness of the results, a peptide was considered as present when detected with at least one spectrum in all the four replicates and absent when not detected in none of the four replicates of a strain.

The proteins identified as included in the phosphoproteome have been blasted against the non-redundant NCBI database (update 2018/07/07) for a more specific annotation.

## Results

The objectives of this work were, first, to establish the phosphoproteome of *S. thermophilus*, the only GRAS species in the streptococci genus, second, to identify the pathways targeted by phosphorylation and third, to evaluate the role of the only Hanks-type kinase identified in the genome of this species in this process. To achieve these goals, we performed in parallel a global shotgun proteomics and a specific phosphoproteomics analyses on both the wild type strain and its Hanks-type kinase deletion mutant. These two types of analyses were necessary to be able to identify peptides whose phosphorylation level but not abundance varied.

### *In silico* Screening of the Whole Genome of *S. thermophilus* LMD9 and Identification of Structural Homologs to PknB

Our *in silico* screening of the entire genome of LMD9 led to the detection of three sequences with a threshold above 95% probability to share the PknB kinase fold, a size above 100 residues and an *e*-value below 1.e^−15^. These are WP_011681404.1 (PknB), WP_011681723.1 (STER_1920; STER_RS09410), and WP_011681158.1 (STER_1047; STER_RS05195) with scores of probability of 100, 99.97, and 99.43%, sequence size of 271, 251, and 138, and e-value of 4.8 e^−45^, 2.9 e^−35^, and 2.2 e^−17^, respectively. Expectedly, the first sequence showing 40% sequence identity with the reference fold is PknB from *S. thermophilus*, despite being annotated as hypothetical protein. This sequence was homology modeled and the combination sequence/structure/function was thoroughly analyzed (see next section). Interestingly the second, which is annotated aminoglycoside phosphotransferase, shows 19% sequence identity, nevertheless among the 719 residues of this putative kinase-like protein, 251 amino acid residues are aligned onto 278 residues of the PknB kinase domain. Notably, the sequence evidences an additional N-terminal domain of 200 amino acid residues. The HMM detection shows that this protein WP_011681723.1 associates the prediction of kinase domain with a putatively conserved catalytic machinery. Accordingly, it displays functional motifs that are degenerated; LEDx_4_N aligns with HRDx_4_N, DLE aligns with DFG, and SQN aligns with SPE, both located at the start and end of the activation loop, respectively. Markedly, the three catalytic residues that belong to HRDx_4_N and DFG motifs are strictly conserved with PknB_STh_ Asp331, Asn336, and Asp349 that correspond to PknB_Mtb_ Asp138, Asn143, and Asp156, respectively. Eventually, this kinase-like portion of WP_011681723.1 could display an activation loop, which contains several Ser and Thr residues known to accept phosphorylation (Supplementary Figure S1). All these elements strongly suggest that this domain could act as a kinase and should be able to phosphorylate substrates, notably in absence of PknB. The third and last sequence identified is WP_011681158.1, annotated as AarF/ABC1/UbiB kinase family protein, which shows 25% sequence identity with PknB_Mtb_. Similarly, this sequence, despite displaying large insertions in the putative kinase domain, contains nevertheless conserved aspartate and asparagine catalytic residues embedded into the degenerated functional motif HGDx_4_N instead of HRDx_4_N and the conserved aspartate of the DFG motif (Supplementary Figure S2). The structural homology and annotation to kinase family as well as the highly conserved catalytic residues suggest that this protein could act as a kinase and could be able to phosphorylate Ser/Thr residues. This result should deserve further assessment.

### *In silico* Structural Characterization Showed That *S. thermophilus* PknB Kinase Exhibits All the Signatures of a Hanks-Type Kinase

**FIGURE 2:**
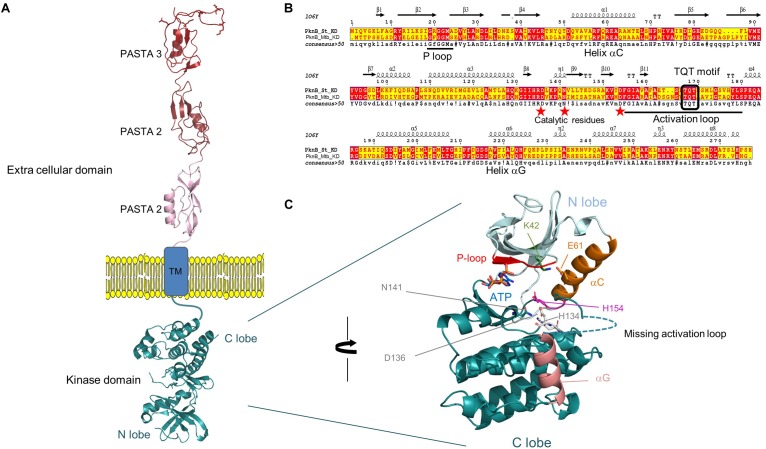
**(A)** Topology of PknB_St_ full length, kinase and extra cellular domain (ECD) have been modeled by homology using PknB, and PASTA coordinates from *M. tuberculosis* and PASTA coordinates of *S. aureus*. Only the TM domain lacks solved structure and could not be modeled. The PASTA domains are shown extended across the peptidoglycan layer. **(B)** ESPript alignment of the kinase domain between PknB from *S. therm*ophilus and *M. tuberculosis*. The structural elements that sign up the hallmarks of Hanks type kinase are conserved and reported in the sequence: helices αC, αG, P- and activation loops. Red stars evidence the catalytic residues. **(C)** Close view of the kinase domain, with the accommodation of an ATP molecule docked in the active site. The structural and functional elements are highlighted: P-loop in red, αC in orange, its residue E61 forming a salt-bridge with K42 that locks αC in a conformation and participates to create a competent active site are in stick, the active triad composed of H134, H138, and N141 are also shown as sticks. Helix αG is in salmon and the missing activation that could not be modeled because too long and with no reference coordinates is drawn as dotted line. The figure has been prepared using PyMOL2.2 Schrödinger.

**FIGURE 3:**
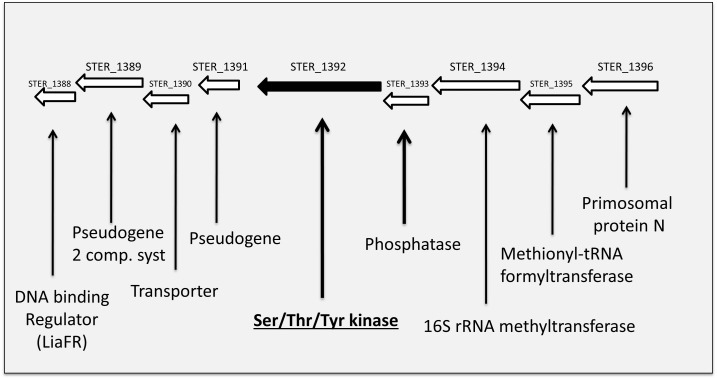
Schematic view of the genetic environment of the PknB encoding gene in the *S. thermophilus* LMD-9 genome.

The Hanks-type serine/threonine kinase PknB from *S. thermophilus* exhibits a classical organization. First, a N-terminal part containing the protein kinase ATP-binding region and the kinase active site. At the C-terminal part, three extracellular PASTA domains interact with peptidoglycan. Between, a transmembrane segment separates the intracellular part from the extracellular part of PknB (Figure 2A). This sequence is well conserved in similar enzymes from different streptococci species (43–58% of identity with PknB from *S. agalactiae*, *Streptococcus mutans*, *S. pneumoniae*, and *S. suis*), except the number of PASTA domain which is higher (four) in *S. pneumoniae* and *S. suis* and only three in the three other species. A gene encoding a serine/threonine phosphatase (STER_1393) is the close neighbor of *pknB*. The two genes are predicted to belong to the same operon ^6^ (Figure 3).

The homology modeling of PknB-KD_St_ catalytic domain displays the extremely conserved protein kinase fold with a N-terminal lobe mainly composed of β-sheet plus the regulatory α-C helix, and the C-terminal lobe that contains almost exclusively helices. The model shows an active and closed conformation that is able to accommodate the cognate ATP molecule in the dedicated pocket, located at the interface between the N and C-lobes. It includes the conserved glycine residues of the phosphate loop (P-loop), the strictly conserved salt-bridge between Lys42 and helix α-C Glu61 that locks the kinase into an active conformation, the DFG motif and the catalytic triad composed of His134, Asp136, and Asn141 (Figure 2B,C). Notably, PknB-KD_St_ contains two main features very close to the bacterial prototypical Ser/Thr kinase PknB-KD_Mtb_ that both deal with the activation loop. The first trait is a similarly long activation loop of 23 residues, starting right after the conserved DFG motif and ending with the conserved SPE motif. As this activation is highly floppy and is hardly seen in crystal structure of template, it could not be modeled. Notably, such flexibility is expected to correlate to versatility of the kinase to tune its binding to the structurally unrelated substrates. This is in total agreement with the 10 highly dissimilar substrates identified in our study. The second feature is a double phosphorylation site contained in the TQT motif located in the middle of the activation loop. PknB-KD_Mtb_ has been shown to require the two threonine to be phosphorylated for a fully active kinase. Possibly, this could be also a regulation trait of PknB_St_ shared with PknB_Mtb_, but this need to be documented. The homology modeling of the extra cellular domain of PknB_St_ displays three PASTA repeats of roughly 70 residues each. In *S. pneumoniae*, these PASTA123 repeats have been shown to behave as interchangeable modules that are nevertheless required to trigger the kinase activity and to control the septal cell wall thickness (Zucchini et al., 2018). In contrast, the distal PASTA moiety drives PknB localization at the division septum and displays a specific motif critical for cell separation. Clearly, this fourth PASTA is missing in *S. thermophilus* and the third one does not display the specific motif expected to interact with the cell wall hydrolase LytB (Zucchini et al., 2018). Markedly, while the shorter C-terminal extremity (44 residues) shares no sequence homology with any PASTA module, this segment shows a very low complexity of amino-acids, with 19 and 10 serine and threonine residues, respectively. Possibly, due to this low complexity, this portion could be intrinsically disordered. Its role in cell division and position at the distal extremity of the extracellular domain remain to be deciphered.

### The *S. thermophilus* Phosphoproteome Concerns Diverse Central and Essential Functions

Our phosphoproteome analysis identified 106 proteins and 410 phosphopeptides corresponding to 161 peptide sequences with different phosphosite positions (Supplementary Table S1 for complete phosphoproteomic data and Table 2 for phosphorylated protein identification and functional categories). The phosphorylation occurred for 43% on serine, 33% on threonine and 23% on tyrosine. In the phosphoproteome, we identified only a few multi-phosphorylated peptides. Those carrying three phosphorylations were found in four proteins: the tyrosine kinase (STER_1393, WP_011681174.1), the carboxyl carrier protein (STER_0435, WP_002946351.1), the UDP-glucose 4-epimerase (STER_1369, WP_011681394.1) and a protein of unknown function (STER_0283, WP_002945999.1) in both strains. No peptide phosphorylation was confidently detectable before the enrichment procedure indicating that the level of peptide phosphorylation is low.

**Table 2:** List of *S. thermophilus* proteins in which phosphorylated peptides have been identified.

Protein short name	Description	YP Protein Number	New WP non-redundant protein number sequence number	*S. thermophilus* LMD9 old locus number	*S. thermophilus* LMD9 new locus number
**Amino acid metabolism and transport**					
LivG	ABC transport system ATP-binding protein	YP_819896.1	WP_002949740.1	STER_0401	STER_RS01955
GdhA	Glutamate dehydrogenase/leucine dehydrogenase	YP_819946.1	WP_011680803.1	STER_0467	STER_RS02295
GlnQ	Amino acid ABC transporter ATP-binding protein	YP_820506.1	WP_011681207.1	STER_1117	STER_RS05525
TrpD	Anthranilate phosphoribosyl transferase	YP_820900.1	WP_011681519.1	STER_1552	STER_RS07630
AroG1	3-deoxy-D-arabino-heptulosonate 7-phosphate (DAHP) synthase	YP_821042.1	WP_002947699.1	STER_1703	STER_RS08325
GlnA	Glutamine synthetase	YP_821087.1	WP_011681641.1	STER_1751	STER_RS08555
**Carbohydrate metabolism and transport**					
Pgi	Glucose-6-phosphate isomerase	YP_820037.1	WP_011680683.1	STER_0241	STER_RS01180
Tkt	Transketolase	YP_819848.1	WP_011680734.1	STER_0349	STER_RS01700
CcpA	Catabolite control protein A	YP_820134.1	WP_004197422.1	STER_0679	STER_RS03345
Eno	Enolase	YP_820138.1	WP_011680939.1	STER_0684	STER_RS03365
GlgP	Glycogen/starch/alpha-glucan family phosphorylase	YP_820422.1	WP_011681138.1	STER_1016	STER_RS05015
GpmA	Phosphoglycerate mutase	YP_820559.1	WP_011226119.1	STER_1172	STER_RS05785
Pyk	Pyruvate kinase	YP_820550.1	WP_011681242.1	STER_1163	STER_RS05740
PgmA	Phosphoglucosamine mutase	YP_820614.1	WP_011226155.1	STER_1230	STER_RS06065
PtsI	Phosphoenolpyruvate protein phosphotransferase	YP_820626.1	WP_011681297.1	STER_1242	STER_RS06130
PtsH	Phosphocarrier protein HPr	YP_820627.1	WP_002946351.1	STER_1243	STER_RS06135
Ldh	L-lactate dehydrogenase	YP_820638.1	WP_011226177.1	STER_1257	STER_RS06205
GapA1	Glyceraldehyde 3-phosphate dehydrogenase	YP_821096.1	WP_011681649.1	STER_1761	STER_RS08615
Fba	Fructose/tagatose biphosphate aldolase	YP_821189.1	WP_011681711.1	STER_1876	STER_RS09185
LacZ	Beta-galactosidase	YP_820735.1	WP_011226267.1	STER_1366	STER_RS06725
LacS	PTS sugar transporter subunit IIA	YP_820736.1	WP_011681393.1	STER_1367	STER_RS06730
GalM	Galactose mutarotase	YP_820737.1	WP_011226269.1	STER_1368	STER_RS06735
GalE	UDP-glucose 4-epimerase	YP_820738.1	WP_011681394.1	STER_1369	STER_RS06740
PpnK	NAD kinase	YP_820780.1	WP_014608552.1	STER_1422	STER_RS06995
RgpA	Glycosyltransferase family 1	YP_820795.1	WP_011681439.1	STER_1437	STER_RS07070
AmyL	Alpha-amylase	YP_820851.1	WP_011681482.1	STER_1500	STER_RS07380
Pfl	Pyruvate formate lyase	YP_820965.1	WP_011226473.1	STER_1622	STER_RS07960
ScrA	Sucrose PTS transporter subunit EIIBCA	YP_821049.1	WP_011681617.1	STER_1710	STER_RS08355
GalU	UDP-glucose-pyrophosphorylase	YP_821134.1	WP_002953557.1	STER_1810	STER_RS08845
**Cell cycle control**					
GpsB	Cell division protein	YP_819787.1	WP_011680702.1	STER_0280	STER_RS01360
PabC	Transporter/YceG motif involved in septum cleavage	YP_819793.1	WP_011680707.1	STER_0288	STER_RS01395
FtsW	FtsW/RodA/SpoVE family cell cycle protein	YP_819997.1	WP_002945261.1	STER_0523	STER_RS02565
FtsA	Cell division protein	YP_820223.1	WP_011225777.1	STER_0776	STER_RS03805
FtsZ	Cell division GTPase	YP_820224.1	WP_011225778.1	STER_0777	STER_RS03810
SepF	Cell division protein	YP_820226.1	WP_011681005.1	STER_0779	STER_RS03820
DivIVA	Cell division initiation protein	YP_820229.1	WP_011681008.1	STER_0782	
RodA	FtsW/RodA/SpoVE family cell cycle protein	YP_820583.1	WP_011681262.1	STER_1197	STER_RS05905
**Cell wall/membrane/envelop biogenesis**					
PcsB	CHAP domain-containing protein	YP_819611.1	WP_011680584.1	STER_0042	STER_RS00210
AcpP1	Acyl carrier protein	YP_819616.1	WP_002949008.1	STER_0048	STER_RS00245
MurN	Aminoacyltransferase/Peptidoglycan interpeptide bridge formation enzyme	YP_820103.1	WP_011680915.1	STER_0644	STER_RS03165
RmlB	dTDP-D-glucose 4,6 dehydratase	YP_820607.1	WP_002947383.1	STER_1222	STER_RS06025
**Energy production and conversion**					
Adk	Adenylate kinase	YP_821196.1	WP_011681712.1	STER_1886	STER_RS09240
**Inorganic ion transport and metabolism**					
Pacl1	Calcium translocating P-type ATPase, PMCA-type	YP_820251.1	WP_011681025.1	STER_0808	STER_RS03975
Cah	Carbonate dehydratase	YP_820966.1	WP_011681555.1	STER_1623	STER_RS07965
**Intracellular trafficking and secretion**					
YajC	Preprotein translocase YajC subunit	YP_819755.1	WP_011225395.1	STER_0243	STER_RS01190
SecA	Preprotein translocase SecA subunit	YP_821044.1	WP_011681614.1	STER_1705	STER_RS08335
**Lipid metabolism**					
AccB	Acetyl-CoA carboxylase, Biotin carboxyl carrier protein	YP_819919.1	WP_011680783.1	STER_0435	STER_RS02130
**Nucleotide metabolism and transport**					
ParE	DNA topoisomerase IV subunit B	YP_820096.1	WP_011680910.1	STER_0633	STER_RS03105
PyrE	Orotate phosphoribosyltransferase	YP_820393.1	WP_011681117.1	STER_0981	STER_RS04840
Apt	Adenine/guanine phosphoribosyltransferase	YP_820576.1	WP_002947203.1	STER_1190	STER_RS05870
LigA	NAD-dependent DNA ligase	YP_820862.1	WP_023909862.1	STER_1513	STER_RS07445
GuaB	IMP dehydrogenase/GMP reductase	YP_821293.1	WP_011227627.1	STER_1992	STER_RS09740
**Post-translational modification, protein turnover, chaperone function**					
GrpE	Nucleotide exchange factor/heat shock protein, chaperonin	YP_819701.1	WP_011680648.1	STER_0162	STER_RS00785
DnaK	Chaperonin	YP_819702.1	WP_011226783.1	STER_0163	STER_RS00790
Tig	Trigger factor	YP_819709.1	WP_011680653.1	STER_0191	STER_RS00935
GroES	Co-Chaperonin	YP_819764.1	WP_002949332.1	STER_0252	STER_RS01225
GroEL	Chaperonin	YP_819765.1	WP_011225399.1	STER_0253	STER_RS01230
PrsA	Foldase	YP_819969.1	WP_011680821.1	STER_0492	STER_RS02415
ClpE	ATP-dependent ClpP protease ATP-binding subunit	YP_820106.1	WP_011680918.1	STER_0648	STER_RS03195
TypA	Translational GTPase involved in stress response	YP_820218.1	WP_011227074.1	STER_0771	STER_RS03780
EpsD	Tyrosine protein kinase	YP_820463.1	WP_011681174.1	STER_1068	STER_RS05305
PknB	Ser/Thr kinase	YP_820752.1	WP_011681404.1	STER_1392	STER_RS06845
ClpL	ATP-dependent Clp protease ATP-binding subunit	YP_820924.1	WP_011681535.1	STER_1578	STER_RS07755
	Asp23/Gls24 family envelope stress protein	YP_821053.1	WP_002951891.1	STER_1714	STER_RS08375
	Asp23/Gls24 family envelope stress protein	YP_821265.1	WP_011681746.1	STER_1962	STER_RS03195
**Translation**					
RplU	50S Ribosomal protein L21	YP_819935.1	WP_002885597.1	STER_0455	STER_RS02235
PapL	CCA tRNA nucleotidyl transferase	YP_819940.1	WP_011680798.1	STER_0461	STER_RS02265
Frr	Ribosome recycling factor	YP_820913.1	WP_002949868.1	STER_0475	STER_RS02335
Tuf	Elongation factor	YP_819998.1	WP_002949971.1	STER_0524	STER_RS02570
RplL	50S Ribosomal Protein L7/L12	YP_820037.1	WP_011680864.1	STER_0568	STER_RS02800
RpsA	30S Ribosomal protein S1	YP_820100.1	WP_011680912.1	STER_0639	STER_RS03135
RpmE	50S Ribosomal protein L31 type B	YP_820233.1	WP_002945948.1	STER_0787	STER_RS03860
PrfA	Peptide chain release factor A	YP_820239.1	WP_002948472.1	STER_0793	STER_RS03890
QueF	NADPH-dependent 7-cyano-7-deazaquanine reductase	YP_820305.1	WP_002946191.1	STER_ 0872	STER_RS04310
RpmC	50S Ribosomal protein L29	YP_821209.1	WP_002952156.1	STER_1899	STER_RS09305
Fus	Elongation factor G	YP_821097.1	WP_011226574.1	STER_1762	STER_RS08620
RpsF	30S Ribosomal protein S6	YP_821065.1	WP_011681624.1	STER_1728	STER_RS08450
RplA	50S Ribosomal protein L1	YP_821124.1	WP_002946412.1	STER_1797	STER_RS08780
Efp	Elongation factor P	YP_821054.1	WP_011227545.1	STER_1715	STER_RS08380
InfA	Translation initiation factor IF-1	YP_821195.1	WP_001040189.1	STER_1885	STER_RS09235
RpmF	50S Ribosomal protein L32		WP_002952208.1	STER_1953	STER_RS09555
RpsD	30S Ribosomal protein S4	YP_821274.1	WP_002952258.1	STER_1973	STER_RS09645
RpoC	DNA-directed RNA polymerase beta subunit	YP_821165.1	WP_011681690.1	STER_1844	STER_RS09010
RplQ	50S Ribosomal protein L17	YP_821190.1	WP_002952134.1	STER_1880	STER_RS09215
RplR	50S Ribosomal protein L18	YP_821201.1	WP_011681714.1	STER_1891	STER_RS09265
RplF	50S Ribosomal protein L6	YP_821202.1	WP_002946167.1	STER_1892	STER_RS09270
RplE	50S Ribosomal protein L5	YP_821205.1	WP_002887058.1	STER_1895	STER_RS09285
RplV	50S Ribosomal protein L22	YP_821212.1	WP_002887063.1	STER_1902	STER_RS09320
RplB	50S Ribosomal protein L2	YP_821214.1	WP_002952161.1	STER_1904	STER_RS09330
**Function unknown or putative**					
	Hypothetical protein	YP_819789.1	WP_011680704.1	STER_0283	STER_RS01370
	DNA-binding protein	YP_820441.1	WP_002946488.1	STER_0353	STER_RS01720
	Hypothetical	YP_819930.1	WP_011680791.1	STER_0449	STER_RS02200
	Hypothetical Sulfur transferase/rhodanese domain-containing protein	YP_820053.1	WP_011680879.1	STER_0588	STER_RS02890
	Hypothetical	YP_820069.1	WP_011226984.1	STER_0605	STER_RS02975
HlyIII	Putative hemolysine III like	YP_820082.1	WP_011680900.1	STER_0618	STER_RS03030
	LytR family transcriptional regulator	YP_820152.1	WP_011680948.1	STER_0698	STER_RS03435
	DUF948 domain-containing protein	YP_820173.1	WP_011680965.1	STER_0721	STER_RS03545
	Hypothetical/DUF3270 domain-containing protein	YP_820175.1	WP_002945999.1	STER_0723	STER_RS03555
	Hypothetical transpeptidase	YP_820213.1	WP_011680998.1	STER_0765	STER_RS03750
	BMP family ABC-transporter periplasmic substrate binding component	YP_820291.1	WP_002950498.1	STER_0856	STER_RS04220
YufQ	ABC transport system, permease component	YP_820294.1	WP_011681056.1	STER_0859	STER_RS04235
HstH	HU family DNA-binding protein	YP_820562.1	WP_002950996.1	STER_1175	STER_RS05800
	Hypothetical DegV protein	YP_820564.1	WP_011681251.1	STER_1178	STER_RS05815
RpoC	Putative dihydroxyacetone kinase	YP_821175.1	WP_011681697.1	STER_1854	STER_RS09055
	DUF965 domain-containing protein	YP_821243.1	WP_011681731.1	STER_1937	STER_RS09475
	XRE-family DNA-binding domain protein	YP_821288.1	WP_011681760.1	STER_1987	STER_RS09715

*The proteins are classified in functional groups. The protein annotation was automatically performed as described in the Section “Materials and Methods.” The proteins in gray are those that have been identified as PknB targets (see Table 3).*

The phosphorylation especially targeted proteins involved in essential pathways (Table 2). In order of diminishing importance, we found, first, many phosphorylated peptides belonging to proteins necessary for translation including 15 ribosomal proteins. The second main group concerns proteins involved in carbon metabolism including glycolysis enzymes, PTS carbohydrate transporters and the catabolite control regulator. We merged 16 proteins with unknown or putative functions in a third group that contain a few putative regulators or DNA-binding proteins. A group of proteins involved in cell cycle control, especially cell division proteins, was also found phosphorylated. Chaperonin and stress response proteins constituted another important group of phosphorylated proteins. Finally, we identified proteins involved cell wall/membrane/envelope biogenesis, intracellular trafficking and secretion, inorganic ion, lipid and amino acids metabolisms and energy production and conversion. Both the serine/threonine (PknB) and the putative tyrosine kinases contained phosphorylated peptides. Only one PknB amino acid (threonine 291) was found phosphorylated in the four WT replicates. However, because the modeling step identified a TQT motif that needs to be phosphorylated for a fully active kinase in *M. tuberculosis* and that is conserved in *S. thermophilus*, we specifically looked at this part of the protein. In our dataset, we identified one peptide including this TQT motif and that was found phosphorylated but only in one replicate and was therefore not retained with our stringent parameters. It, however, indicates that this position might be also phosphorylated in *S. thermophilus*. Moreover, it has to be noticed that 6 proteins in which phosphorylation sites have been detected were not identified in the proteome data set probably because of their low abundance.

### The *pknB* Gene Disruption Clearly Affects the Phenotype and the Phosphoproteome of *S. thermophilus* but Not Its Proteome

**FIGURE 4:**
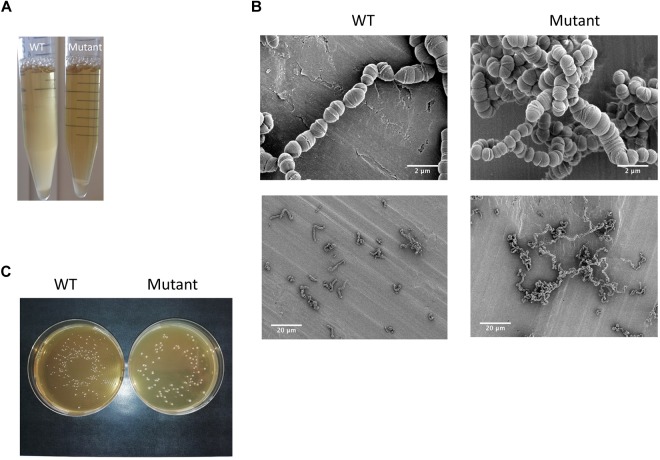
**(A)** Visual and **(B)** microscopic observation at two degrees of magnification of the wild type strain and the Δ*pknB* mutant grown in M17 lactose medium; **(C)** colonies phenotypes of the wild type and the mutant on M17 lactose agar medium.

**FIGURE 5:**
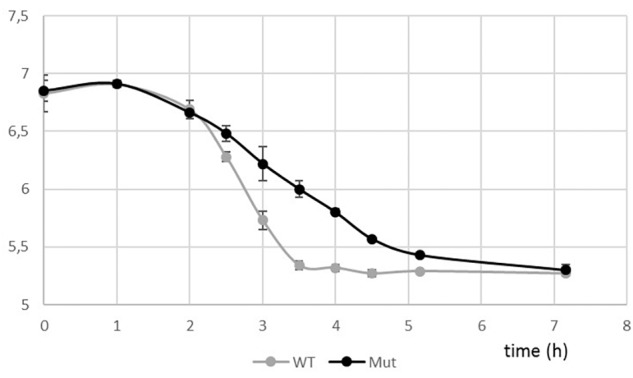
pH evolution curves during growth of the wild type and the Δ*pknB* strains in M17 lactose medium.

Taking advantage of the natural competence of the *S. thermophilus* strain used, we easily replaced the *pknB* gene by an antibiotic cassette. The phenotype of the mutant during growth in liquid medium was visually different from the one of the wild type. A higher sedimentation of the bacteria was observed with the mutant (Figure 4A). Microscopy observation revealed that the PknB bacteria form aggregates in addition to longer chains (Figure 4B). We also observed clear cell division defects. After growth on plates, mutant colonies appear bigger that the wild type ones, which is in agreement with longer chains in the mutant (Figure 4C).The two strains grew well in rich medium, but it was difficult to precisely compare the growth rates of the two strains using absorbance measurements due to culture heterogeneity of the mutant. We therefore followed pH evolution of the two strains during growth in M17 lactose medium (Figure 5). pH decrease was delayed in the mutant but reached the same value than the wild type, i.e., 5,3 at the end of exponential phase.

We evaluated the survival of the two strains after a −20°C freezing stress. It was not possible to compare the bacterial counts of the two strains since their chain lengths were different. We therefore calculated for each strain the percentage of cells that growth on plate after freezing without any additive. Unexpectedly, the mutant exhibited a higher survival rate (73%) than the wild type strain (9%). Longer chains could have a protective effect against a freezing stress.

The proteome analysis, realized in one culture condition (exponential phase in rich M17 lac medium) on a total bacterial extract, allowed to identify 891 proteins on the basis of at least two peptides per protein (listed in Supplementary Table S2) representing half of the theoretical proteome. Based on spectral counts, no statistically significant difference in protein abundance was found in our data set except for PknB that was no more detected, as expected, in the mutant. Among the proteins identified, 82% are predicted to be cytoplasmic (LocateP, Zhou et al., 2008) while the others are associated in different manners to bacterial envelopes. The PknB encoding gene is predicted to be in operon with a gene encoding a phosphatase (STER_1393, WP_011681405.1) ^7^ (Figure 3) which is present in the proteomes of the two strains. Another putative protein kinase, annotated tyrosine kinase (WP_011681174.1) is also present in the proteomes. Although they were clearly identified and quantified, these two kinases and the phosphatase were not at the top of the most abundant proteins identified.

Proteome and phosphoproteome were realized in parallel with mutant and wild type strains. Differences in protein phosphorylation, which were not imputable to protein abundance differences, were highlighted. Concerning the phosphorylation, we identified phosphorylated peptides from 9 proteins that showed presence/absence variations between the wild type and mutant strains in the four replicates (Table 3 and Supplementary Figure S3). We can conclude that at least these 9 proteins plus probably PknB itself, are, direct or indirect, targets of PknB. However, many other peptides remain fully or partially phosphorylated in the mutant (Supplementary Table S3) indicating that other kinase(s) than PknB participate in the protein Ser/Thr/Tyr phosphorylation process.

**Table 3:** List of phosphorylated proteins that are no more phosphorylated in the Δ*pknB* mutant in the four replicates bases on spectral counts.

Protein short name	Protein name	Non-redundant protein ID	STER number	Phosphopeptides	Occurrence of peptide phosphorylation (WT/Mut) Means of spectral counts
FtsA	Cell-division protein	WP_011225777.1	STER_0776	IPVEN**T**VEVPQPVDGENHEQK (**T** 427)	7/0
SepF	Division protein	WP_01168100.1	STER_0779	SDVQK**T**QVLR (**T** 68)	7/0
DivIVA	Cell division initiation protein	WP_011681008.1	STER_0782	NLNE**T**QTFK/LNISE (**T** 282) VLDEHVPDSNDAA**S**FDA**T**R (**S** 197 or **T** 201)	25/0 14.5/0
GroEL	Chaperonin	WP_011225399.1	STER_0253	APAAPATDPGMMoxG**Y** (**Y** 539)	7/0
(PcsB)	Hypothetical protein	WP_011680584.1	STER_0283	TQNSYEE**S**QELDFQDAK (**S** 13) KIEADGD**TS**PLDAFIQK (**T** 73 or **S** 74)	17/0 12.5/0
MltG (PabC)	Endolytic transglycosylase	WP_011680707.1	STER_0288	NLSIPQE**T**EILK (**T** 139)	6/0
Fus	Elongation factor	WP_011226574.1	STER_1762	IGE**T**HEGASQMDWMEQEQER (**T** 43)	6/0
RpmC	50S Ribosomal protein L29	WP_002952156.1	STER_1899	FQAAAGQLDQ**T**AR (**T** 47)	8/0
RodZ-like (PurH)	RodZ-like, XRE-family DNA-binding domain protein	WP_011681760.1	STER_1987	**Y**ATSVDLDGK (**Y** 58)	10/0

*Sequences of phosphorylated peptides are given and phosphorylation positions are in bold. These proteins were specifically blasted against the non-redundant NCBI database. In a few cases, the protein identifier is not the same that the ones obtained in the automatic identification process used in Table 2. The latter are given between brackets.*

The 9 proteins containing phosphorylated peptides that were no more phosphorylated in the Δ*pknB* mutant are mainly strongly linked to the division process and located, as PknB, close or within the cytoplasmic membrane, probably at the division site. Four of them (PknB, RodZ, MltG, and Ster_0283) are predicted to contain a transmembrane segment. In addition, RodZ contains a HTH_XRE intracellular motif indicating a possible transcriptional regulatory role for this protein. FtsA, SepF, DivIVA, and RodZ are proteins well-known to be involved, as PknB, in the division process in streptococci as well as in many bacteria (Massidda et al., 2013). The cases of the chaperonin GroEL and the elongation factor Fus are, in a less obvious way, probably linked to PknB and the cell division in *S. thermophilus*. They are indeed known to be phosphorylated and identified as substrates of the Hanks-type kinase PrkC in *Bacillus anthracis*. In this bacterium, the phosphorylation of GroEL is critical for biofilm formation (Arora et al., 2017). GroEL was also found co-localized with FtsZ in *Escherichia coli* suggesting a role in cell division (Ogino et al., 2004). MltG is a protein containing a transmembrane segment. It is described as an endolytic transglycosylase from the YceG family, well-conserved within bacteria and cleaves nascent peptidoglycan polymers and terminates their elongation (Yunck et al., 2016). Finally, the presence of RpmC in the list of PknB targets remains unexplained at this step.

The sites of phosphorylation of the nine proteins targets of PknB have been identified (Table 3). Phosphorylated amino acids were, as expected mainly serines and threonines. Our results also indicate that two phosphopeptides (in GroEL and RodZ proteins), impacted by the absence of PknB, were phosphorylated on tyrosines. Tyrosine phosphorylation by Hanks-type kinases was already observed in *M. tuberculosis* (Kusebauch et al., 2014). We cannot discount the possibility of an indirect effect of PknB via the modification of the tyrosine kinase phosphorylation state. Evidences of cross-phosphorylation processes between Hanks-type and tyrosine kinases have been given by Shi et al. (2014). However, in our data set, we cannot confirm this hypothesis since we did not identify significant modification of the tyrosine kinase phosphorylation state between the two strains.

## Discussion

Several research groups, working with pathogen bacteria, have hypothesized that a phosphoproteome composed of a large number of proteins and a high proportion of multiple phosphorylation sites may be closely related to bacterial virulence (Sun et al., 2010). We demonstrated in this work done on *S. thermophilus*, a bacterium recognized as GRAS and massively used in the dairy industry, that protein phosphorylation is also an important process in non-pathogenic bacteria. Although not directly comparable, the phosphoproteome of this bacterium, established in one culture condition is of the same order of magnitude (around 100 phosphorylated proteins) than the one of *S. pneumoniae* (Sun et al., 2010). Our results are also coherent with those obtained with another main starter bacterium: *L. lactis* (Soufi et al., 2008). The level of phosphorylation is too low to be detected without an enrichment step although a very sensitive last generation mass spectrometer was used. We did not identified a clear recognition motif for phosphorylation and it therefore remains impossible to predict which protein will be phosphorylated and where. In the *S. pneumoniae* phosphoproteome, Sun et al. (2010) did not identify a specific sequence for phosphorylation either and only found that phosphorylation prefers hydrophobic sequences to occur.

We demonstrated that, in *S. thermophilus* as in other bacteria, phosphorylation target central functions sustaining growth such as translation, division and carbon metabolism. Even if these functional categories are globally well conserved, the specific proteins phosphorylated can differ from one set to another one. The sites of phosphorylation of a same protein in different data sets can also vary as observed in *M. tuberculosis* (Calder et al., 2016). Elsewhere, even if their proportion is lower compared to what was noticed in *S. pneumoniae*, we also observed proteins harboring multi phosphorylation sites (up to four in our study). The level of phosphorylated tyrosine observed in *S. thermophilus* is rather high compared to other phosphoproteomes (*S. pneumoniae, L. lactis*, and *Bacillus subtilis*) in which it hardly reaches 10% (Macek et al., 2007; Soufi et al., 2008; Sun et al., 2010).

The impact of *pknB* gene disruption is very clear on cell division and morphogenesis as observed with the naked eye and by microscopy. Longer chain and division defects were observed in the Δ*pknB* mutant. Longer chains were also observed in *S. suis* and *S. agalactiae* Δ*pknB* mutants (Rajagopal et al., 2003; Zhang et al., 2017). In *S. thermophilus*, these observations correlate well with phosphoproteome modifications between the two strains, which affect, in an important manner, proteins involved in cell division process.

Unexpectedly, the proteomes obtained from the *S. thermophilus* wild type strain and the Δ*pknB* mutant did not differ. No protein abundance variation, based on spectral counts, was significant apart from PknB which was completely absent from the mutant data set consequently to the deletion of its encoding gene. We cannot exclude that abundance of some proteins, not visible in our preparation, varied. We indeed worked with a total extract, which do not allow an optimal visualization of all membrane, and cell wall proteins, that are less abundant, compared to cytoplasmic ones in a global extract. A prior fractionation of cell into cytoplasm and envelopes would allow the quantification of more proteins and confirm or not the present result.

Via this work, we brought new data confirming that bacterial Ser/Thr Hanks-type kinase targets bacterial division. We can hypothesize that all proteins, substrates of PknB, are involved in the cell division process and located near the cytoplasmic membrane of PknB. As we did identify neither conserved sequences nor FHA motif in the proteins that are PknB targets, we cannot explain PknB specificity based on primary sequence analysis. We hypothesized that the co-localization of PknB with its substrates could be the main factor driving its specificity. A possible role of the hypothetical protein (STER_0283) and of RpmC in cell division, as well as the identification of genes possibly regulated by RodZ need therefore to be investigated. The phosphorylation of other proteins is also affected by the absence of PknB but do not fully fulfill the stringent criteria fixed. We can mention, as example, the protein FtsW, involved in division and whose peptides were no more phosphorylated in the mutant and phosphorylated in the wild type but not in all replicates.

**FIGURE 6:**
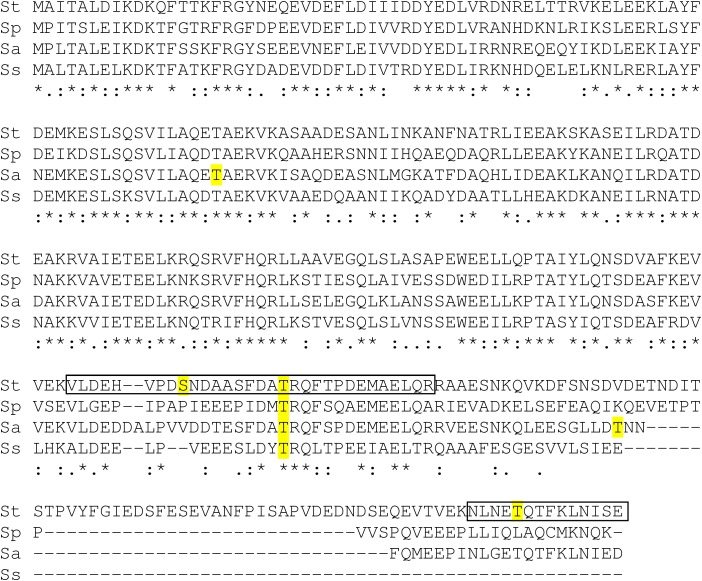
Alignment of DivIVA proteins from *Streptococcus thermophilus* (St), *pneumoniae* (Sp), *agalactiae* (Sa), and *suis* (Ss) and identification of phosphopeptides (framed sequences, this work) and others identified phosphorylated amino acids (highlighted in yellow).

The protein DivIVA, involved in cell division initiation, is the most emblematic substrate of streptococcal Hanks-type kinases. Several phosphopeptides have been identified within streptococcal DivIVA proteins. One containing the threonine 201 (in *S. thermophilus*) seems conserved among the four streptococcal species while others have been identified only in one species and could be more specific (Figure 6). The phosphorylation of the threonine 282 in the C-terminal peptide in *S. thermophilus* was not yet reported and this amino acid is not conserved in *S. pneumoniae*.

As already reported, transcriptional regulators and histidine kinases from two-component systems (TCS), can be regulated via Ser/Thr/Tyr phosphorylation (Kalantari et al., 2015; Khara et al., 2018). In our data set, we identified components of three TCS among the eight present in the strain: the orphan response regulator CovR-like (STER_0354), the whole WalR-like system (STER_1115 and STER_1116), the Lia-like response regulator. None of these proteins was found phosphorylated in our experiment.

As already observed in pathogen streptococci, PknB is only responsible for a minor part of the Ser/Thr/Tyr protein phosphorylation observed. We identified, with a stringent process, 9 proteins substrates of PknB while 7 and 12 proteins were found substrates of homolog kinases in *S. pneumoniae* (Novakova et al., 2010) and *S. suis* (Zhang et al., 2017), respectively. These observations indicate PknB is probably specialized in the phosphorylation of co-localized proteins involved in cell division and that other enzymes are involved in the global phosphorylation process. Of course, these PknB substrates should be confirmed by targeted proteomics approaches. A more specific analysis of an extract enriched in envelope-associated proteins could help to identify additional PknB targets.

It appears clearly from our work that PknB is not responsible for the whole phosphorylation process. We identified two potential candidates showing a kinase structural homology with PknB and whose activity and targets need to be demonstrated and identified, respectively.

## Author Contributions

CH and LH set up and realized proteomics and phosphoproteomics experiments. CH, LH, MZ, and MB-N participated in proteomics data analysis and manuscript. AC realized microscopic observations. MV, RG, CM, and MB were in charge of molecular biology and microbiology experiments, especially the construction of the mutant and its characterization. SS, VMa, and GA-L characterized PknB from a structural point of view, modeled it, and identified other possible kinases. VMo initiated the study, coordinated it, participated in data analysis and interpretation, and wrote the main part of the manuscript.

## Conflict of Interest Statement

The authors declare that the research was conducted in the absence of any commercial or financial relationships that could be construed as a potential conflict of interest.

## Footnotes

1 https://www.ncbi.nlm.nih.gov/genome/420?genome_assembly_id=300120

2 https://toolkit.tuebingen.mgp.de/

3 http://www6.jouy.inra.fr/mima2/

4 http://pappso.inra.fr/

5 http://pappso.inra.fr

6 www.microbesonline.org/operons/gnc322159.html

7 www.microbesonline.org/operons/gnc322159.html

## Supplementary Material

FIGURE S1 The figure shows a clustalW alignement using Espript (http://espript.ibcp.fr/ESPript/ESPript/) which evidences the secondary structure elements of PknB kinase domain *M. tuberculosis* (pdb id code 1O6Y) along with the sequences between PknB kinase domain from *S. thermophilus*, and putative kinase named WP_0116817231.

FIGURE S2 The figure shows a clustalW alignement using Espript (http://espript.ibcp.fr/ESPript/ESPript/) which evidences the secondary structure elements of PknB kinase domain *M. tuberculosis* (pdb id code 1O6Y) along with the sequences between PknB kinase domain from *S. thermophilus*, and putative kinase named WP_0116811581.

FIGURE S3 Phosphopeptides spectra of the protein targets of PknB listed in Table 3.

TABLE S1 Identification of 891 proteins found by LC-MS/MS and present in the 8 samples (4 WT and 4 mutants). This table gives details about peptides and proteins.

TABLE S2 Identification by LC-MS/MS of 161 phosphoproteins present in the triplex with light, medium and heavy dimethyl labeling (48 Samples).

TABLE S3 Spectral counting of phosphopeptides: present/absent in WT/Mutant by sample and genotype.
