# Supplementary figures and images for: Identification of Hanks-Type Kinase PknB-Specific Targets in the Streptococcus thermophilus Phosphoproteome
Source: Front Microbiol. 2019 Jun 19;10:1329. doi: 10.3389/fmicb.2019.01329 (PMC6593474; doi:10.3389/fmicb.2019.01329)

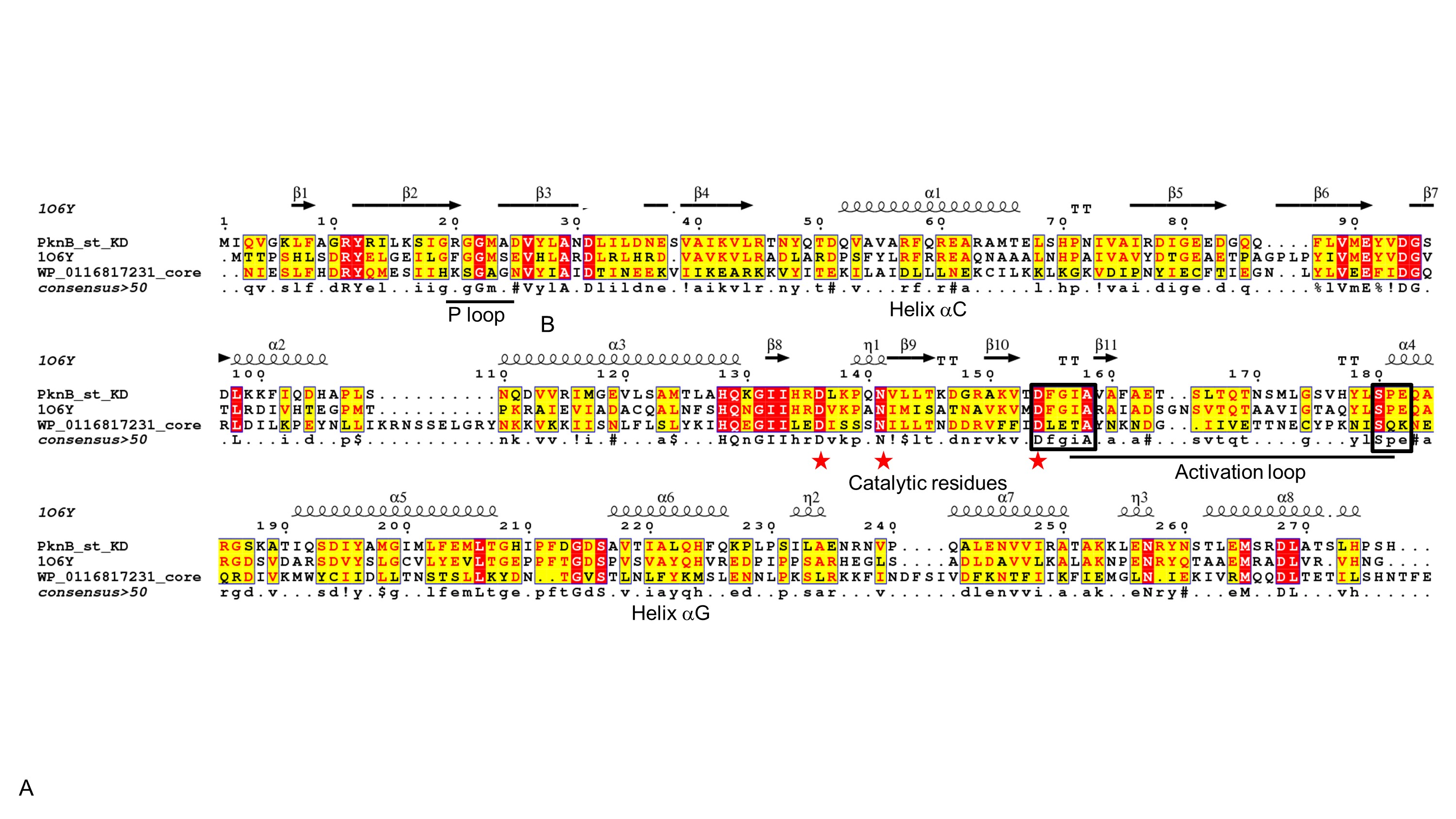

Supplement: FIGURE S1 — The figure shows a clustalW alignement using Espript (http://espript.ibcp.fr/ESPript/ESPript/) which evidences the secondary structure elements of PknB kinase domain M. tuberculosis (pdb id code 1O6Y) along with the sequences between PknB kinase domain from S. thermophilus, and putative kinase named WP_0116817231. [file Image_1.JPEG]

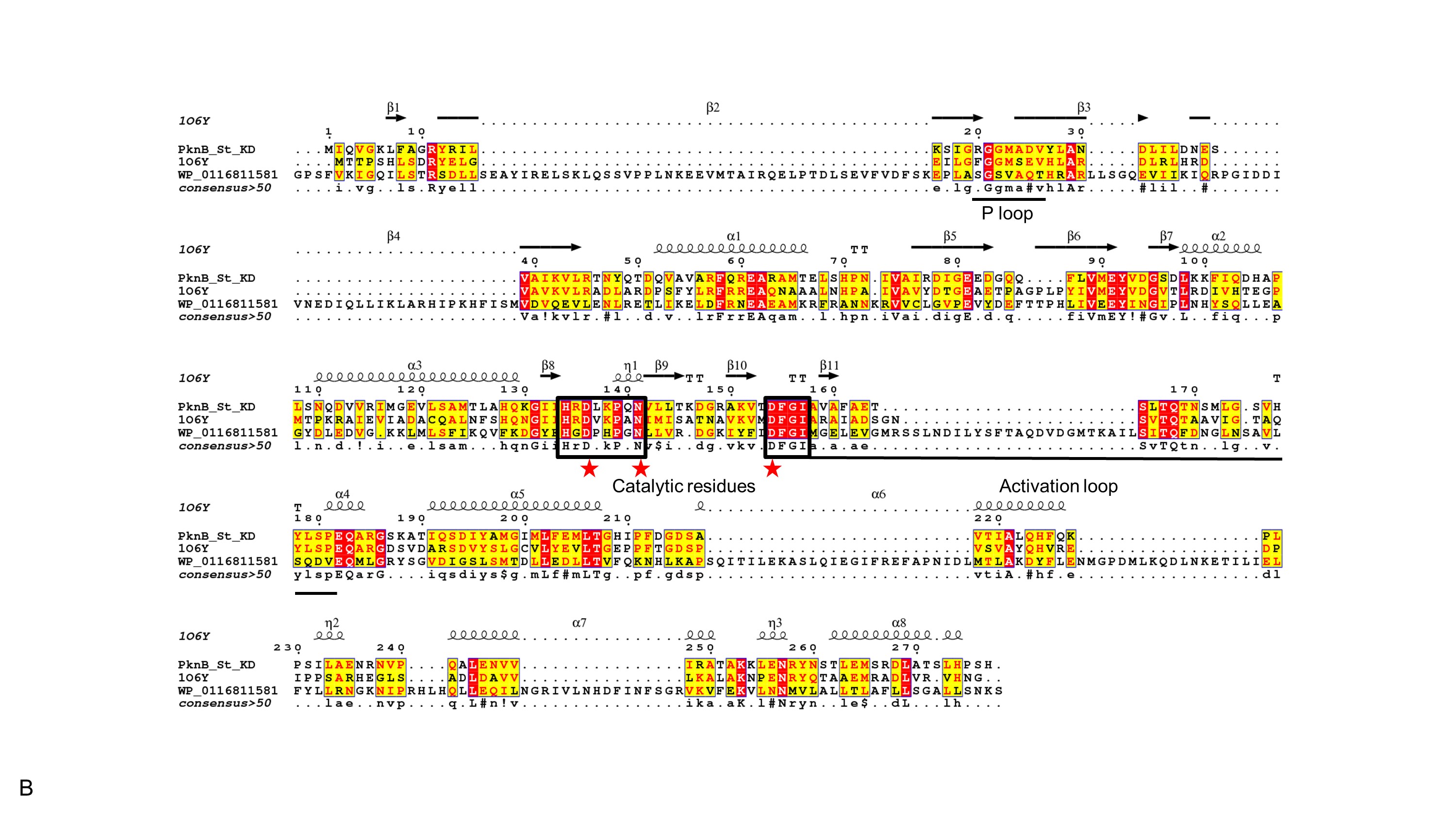

Supplement: FIGURE S2 — The figure shows a clustalW alignement using Espript (http://espript.ibcp.fr/ESPript/ESPript/) which evidences the secondary structure elements of PknB kinase domain M. tuberculosis (pdb id code 1O6Y) along with the sequences between PknB kinase domain from S. thermophilus, and putative kinase named WP_0116811581. [file Image_2.JPEG]
