# Supplementary material for: Identification of Hanks-Type Kinase PknB-Specific Targets in the Streptococcus thermophilus Phosphoproteome
Source: Front Microbiol. 2019 Jun 19;10:1329. doi: 10.3389/fmicb.2019.01329 (PMC6593474; doi:10.3389/fmicb.2019.01329)

FtsA ; STER_0776 ; IPVENTVEVPQPVDGENHEQK (T 427)

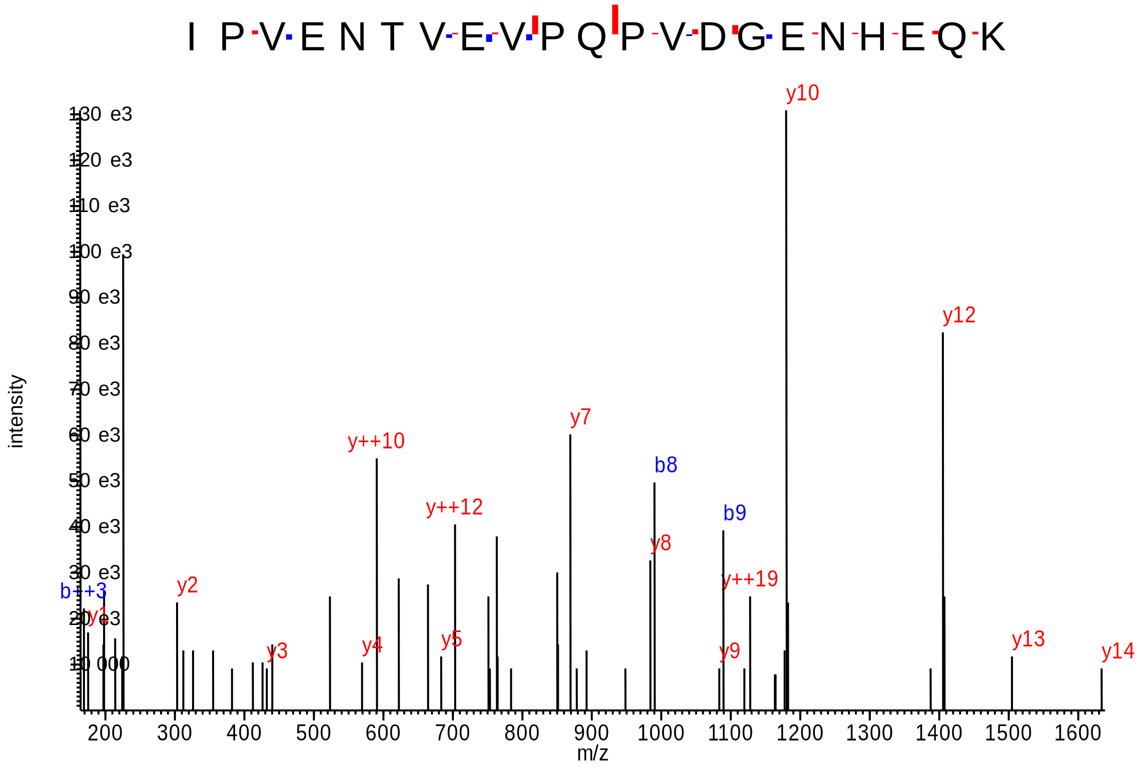

SepF ; STER_0779 ; SDVQKTQVLR (T 68)

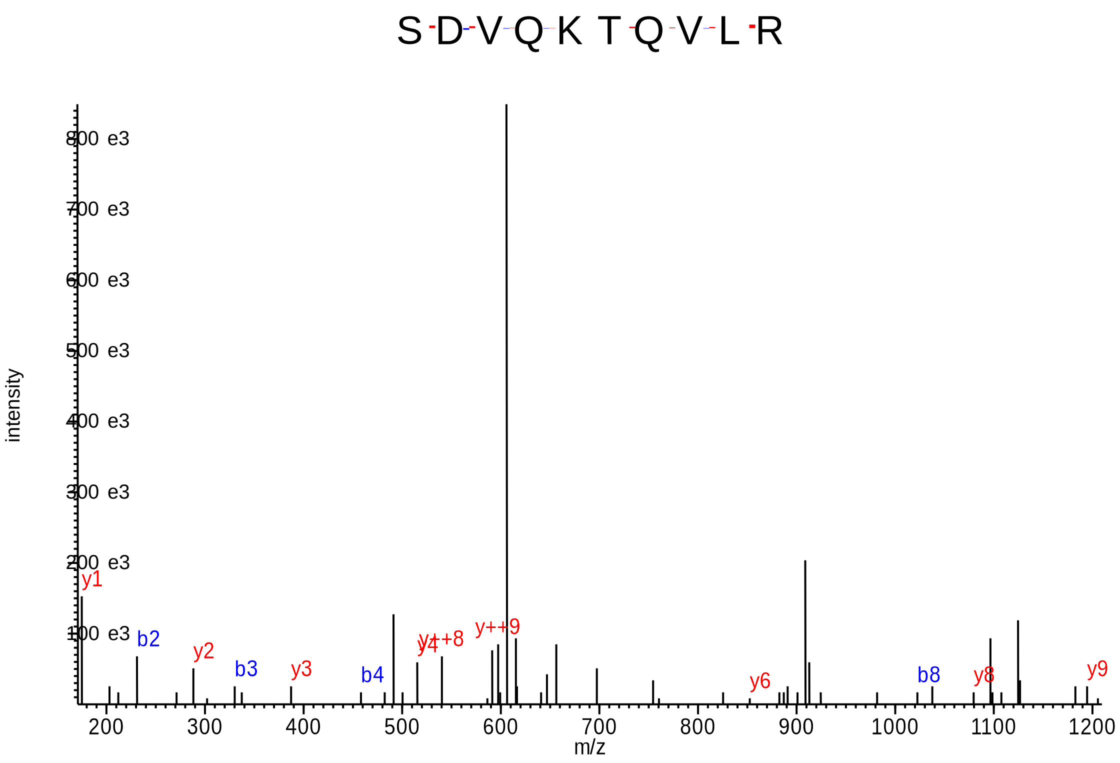

DivIVA ; STER_0782 ; NLNETQTFK/LNISE (T 282)

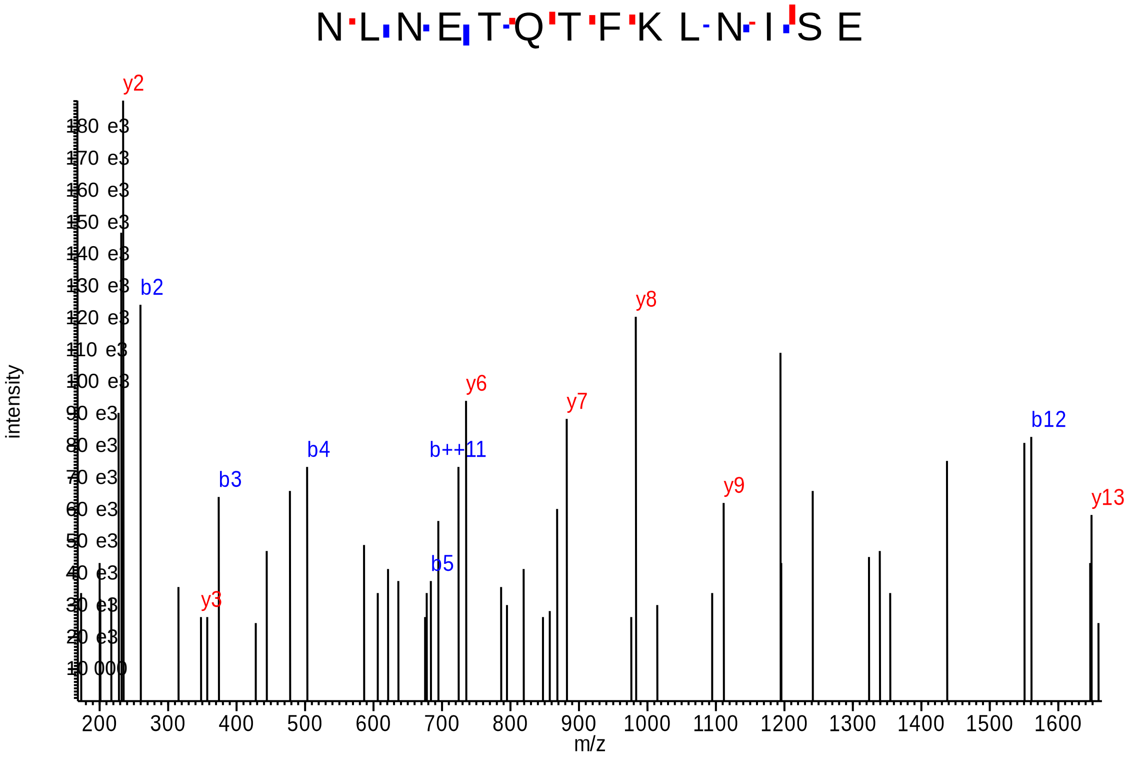

DivIVA ; STER_0782 ; VLDEHVPDSNDAASFDATR (S 197)

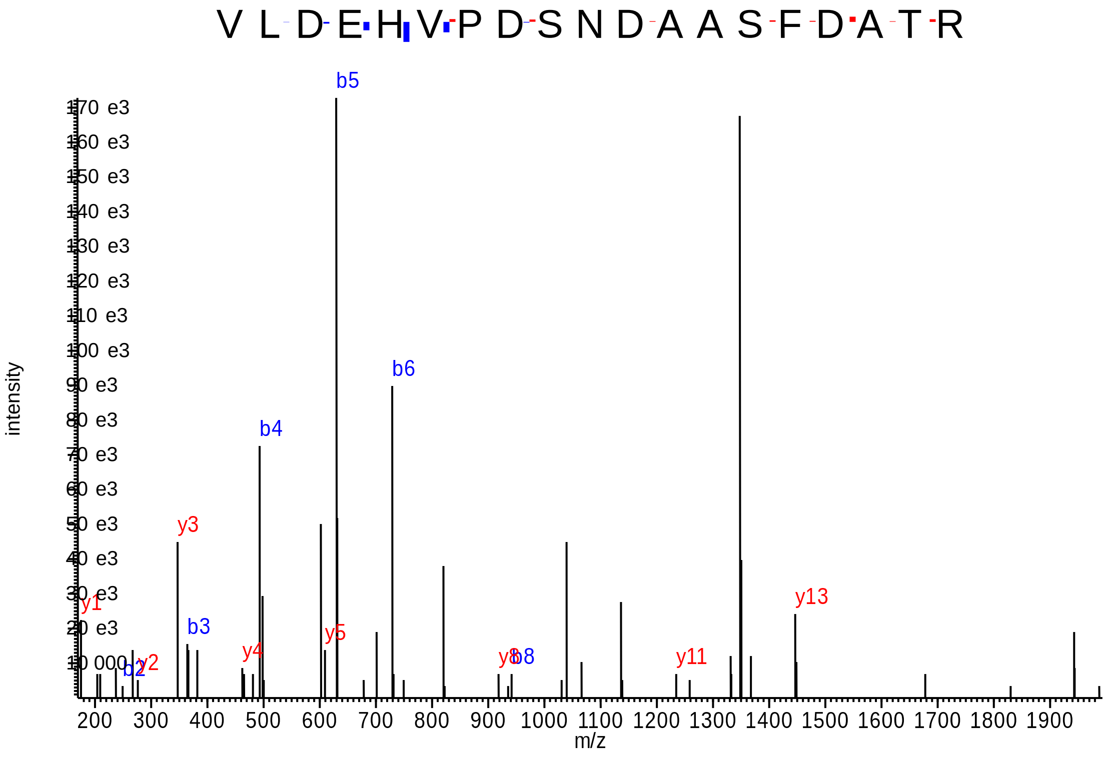

DivIVA ; STER_0782 ; VLDEHVPDSNDAASFDATR (S 201)

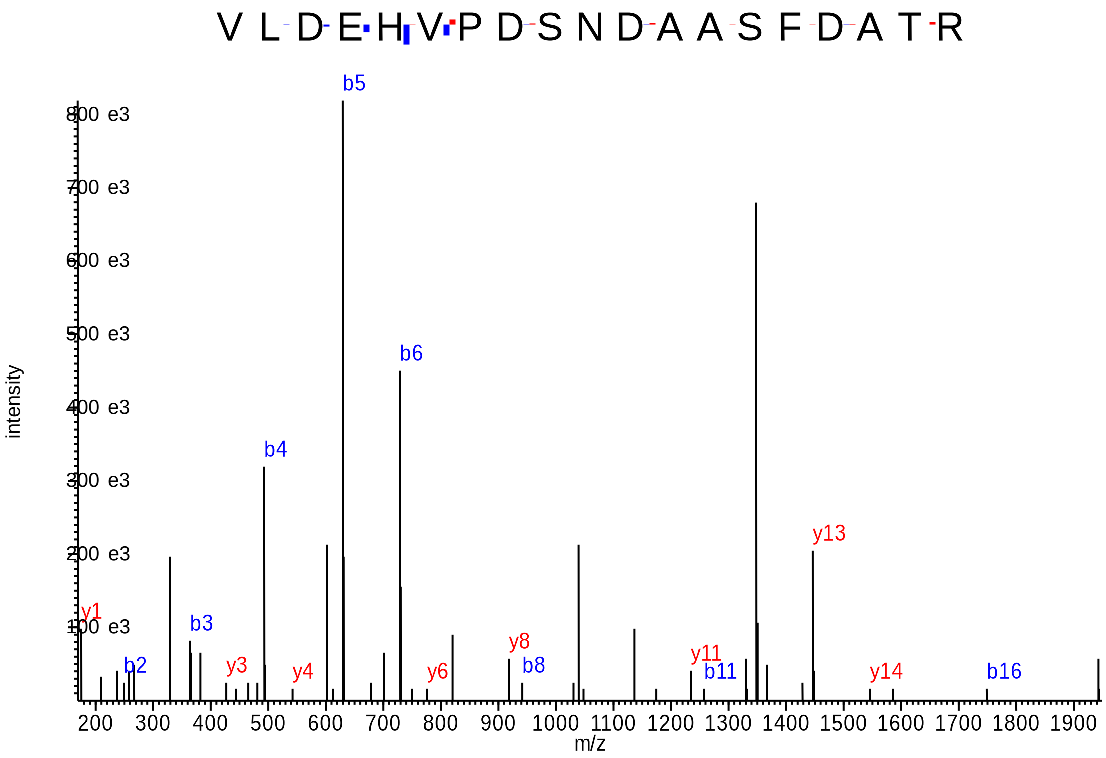

GroEL ; STER_0253 ; APAAPATDPGMMoxGY (Y 539)

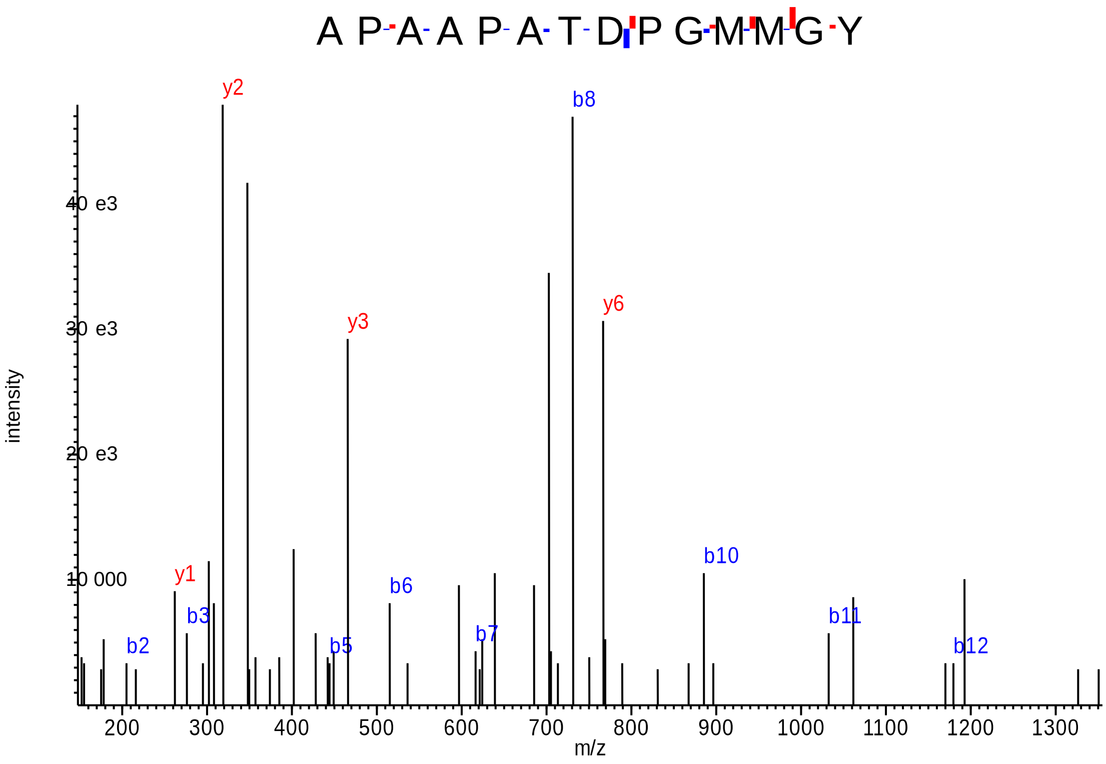

(PcsB) ; STER_0283 ; TQNSYEESQELDFQDAK (S 13)

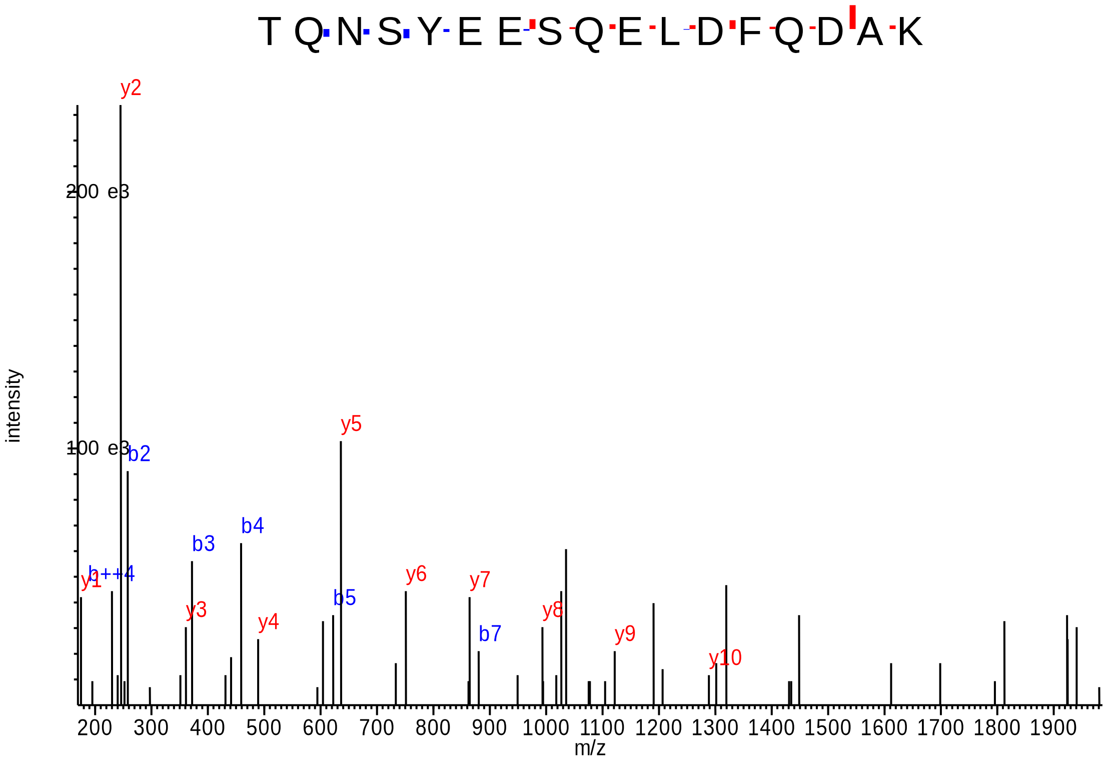

(PcsB) ; STER_0283 ; KIEADGDTSPLDAFIQK (T 73)

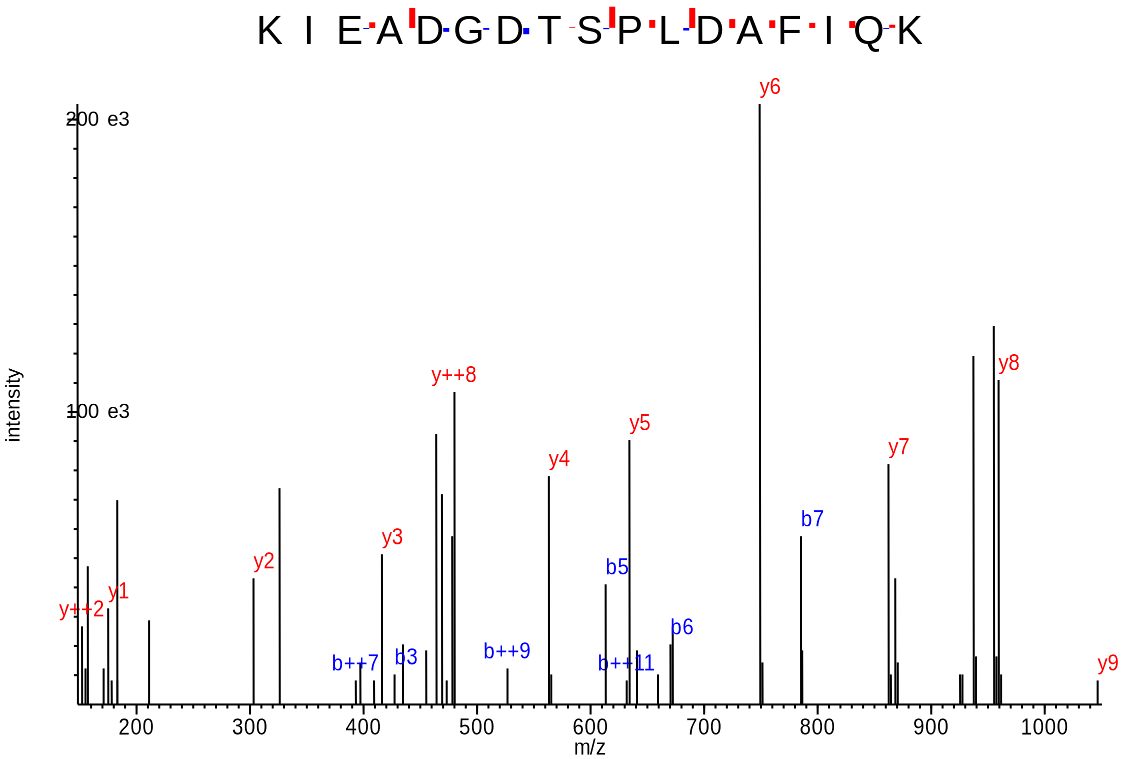

(PcsB) ; STER_0283 ; KIEADGDTSPLDAFIQK (S 74)

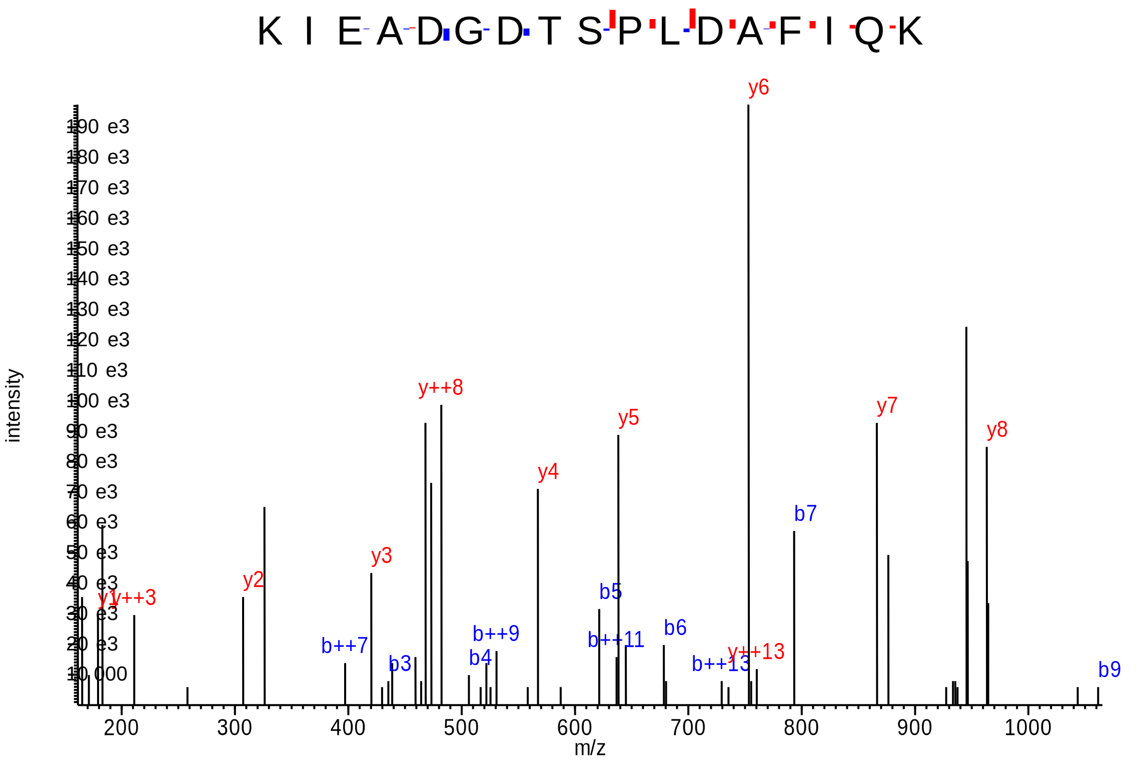

MltG (PabC) ; STER_0288 ; NLSIPQETEILK (T 139)

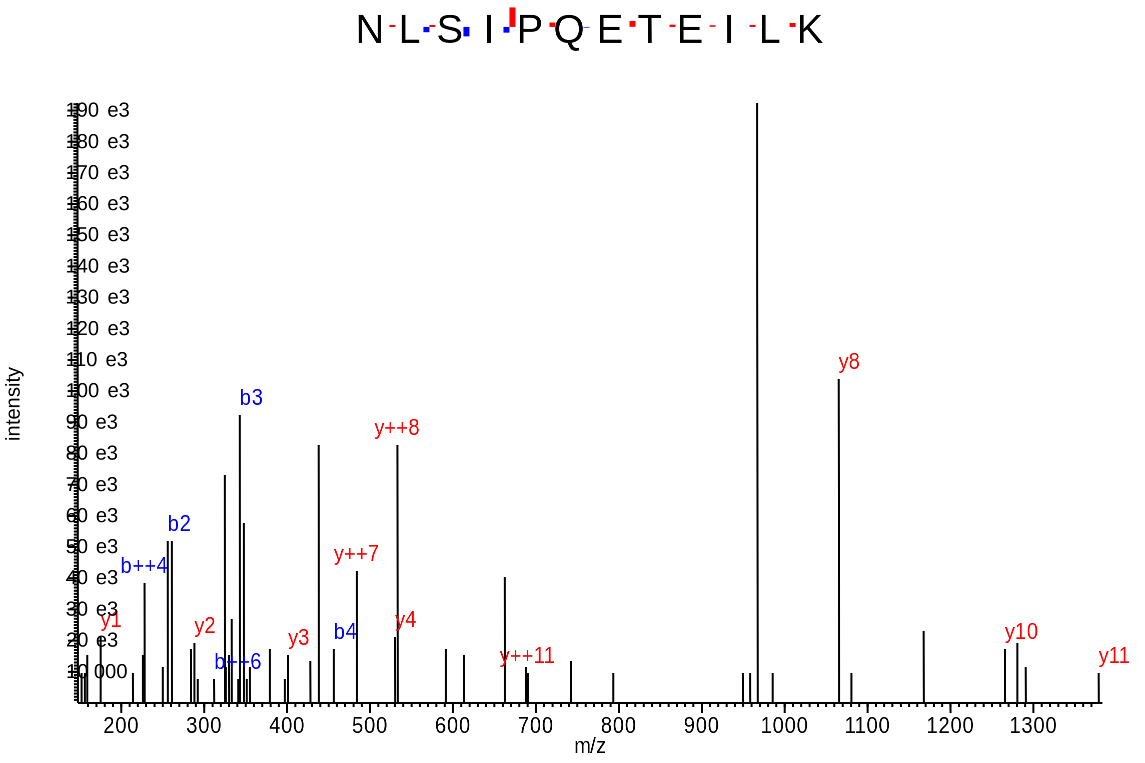

Fus ; STER_1762 ; IGETHEGASQMDWMEQEQER (T 43)

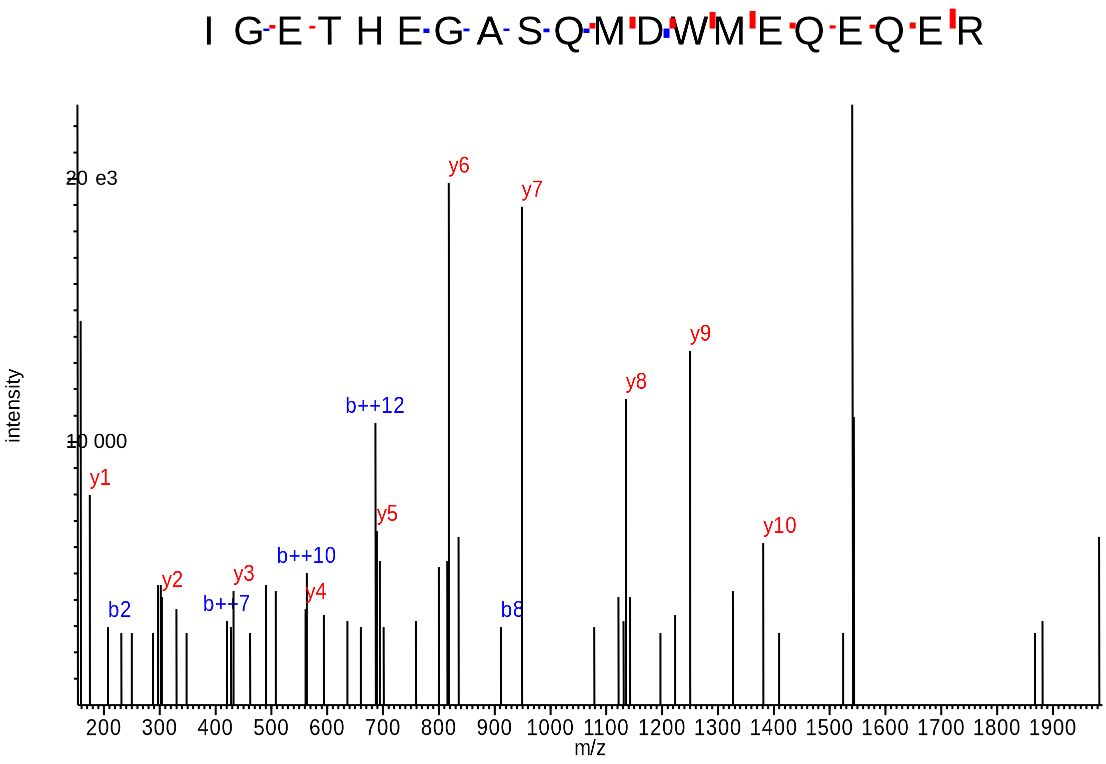

RpmC ; STER_1899 ; FQAAAGQLDQTAR (T 47)

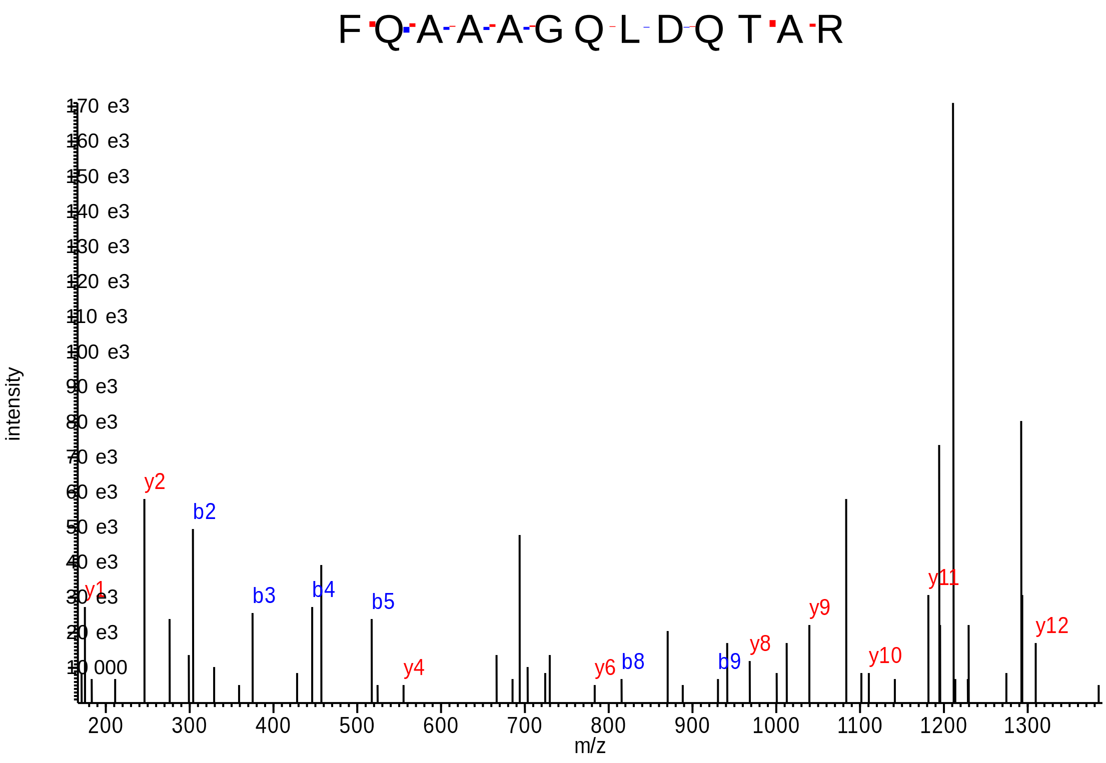

RodZ-like (PurH) ; STER_1987 ; YATSVDLDGK (Y 58)

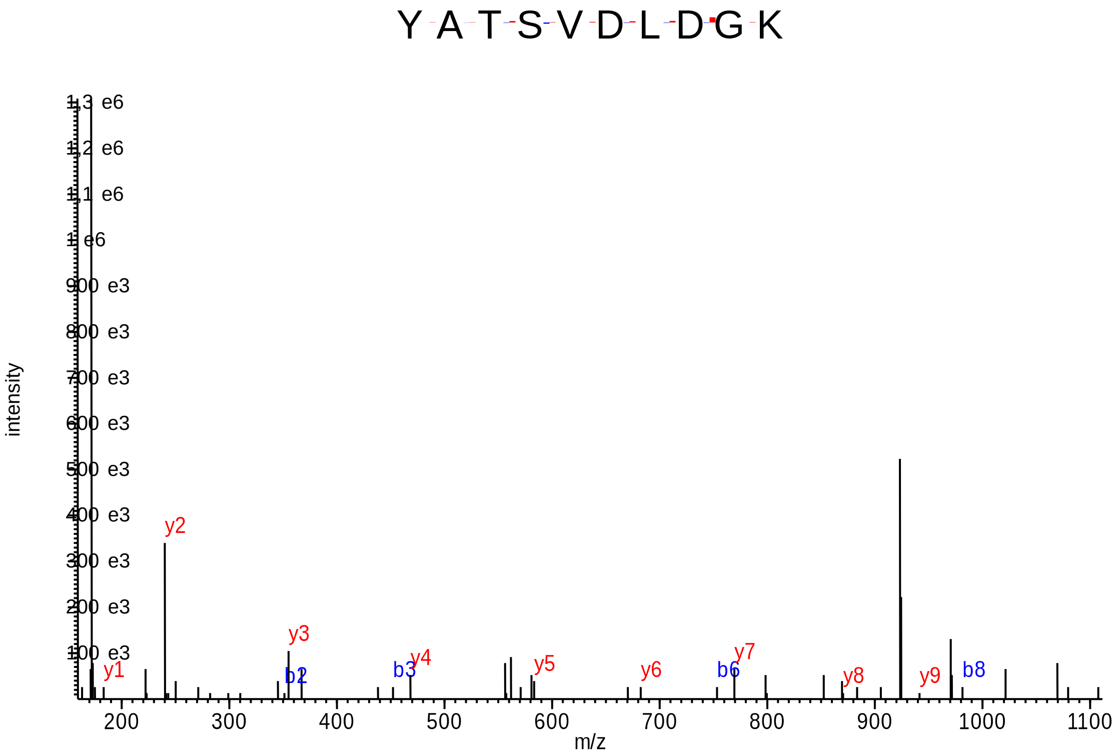

Supplement: FIGURE S3 — Phosphopeptides spectra of the protein targets of PknB listed in Table 3. [file Data_Sheet_1.PDF]
